# Diagnostic performance of ultrasound characteristics-based artificial intelligence models for thyroid nodules: a systematic review and meta-analysis

**DOI:** 10.3389/fonc.2025.1614603

**Published:** 2025-09-03

**Authors:** Jianfeng Zhan, Jian Zhang, Shaoqi Zhu, Lin Ni, Chen Zhang, Jia Hu

**Affiliations:** ^1^ Department of Endocrinology, Huzhou First People’s Hospital, The First Affiliated Hospital of Huzhou University, Huzhou, Zhejiang, China; ^2^ School of Medicine (School of Nursing), Huzhou University, Huzhou, Zhejiang, China; ^3^ Department of Hepatobiliary and Pancreatic Surgery, Huzhou Central Hospital affiliated to Huzhou University, Huzhou, Zhejiang, China

**Keywords:** artificial intelligence, diagnosis, thyroid nodule, thyroid neoplasms, ultrasound

## Abstract

**Background:**

Nowadays, artificial intelligence (AI) diagnostic models based on ultrasound features have been gradually integrated into the evaluation of thyroid nodules. However, the diagnostic effects of different AI-assisted diagnosis methods vary greatly.

**Objective:**

This study aims to systematically evaluate the performance of the ultrasound-based artificial intelligence diagnostic models in differentiating benign and malignant thyroid nodules and to determine the most effective diagnostic model.

**Methods:**

We conducted a comprehensive literature search in PubMed, Web of Science, and the Cochrane Library using subject-specific keywords to identify studies on AI-assisted thyroid nodule diagnosis. Study quality was assessed using Quality Assessment of Diagnostic Accuracy Studies-2 (QUADAS-2). Meta-analysis was performed using Meta-Disc 1.4, Review Manager 5.4, R 4.4.2, and Stata 17.0. Pooled sensitivity, specificity, diagnostic odds ratio (DOR), and area under the summary receiver operating characteristic curve (SROC-AUC) with 95% confidence intervals (CI) were calculated. Subgroup analyses and clinical applicability assessments were conducted.

**Results:**

Twenty-eight studies involving 134,028 patients, 158,161 thyroid nodules, and 529,479 ultrasound images were included. The AI-assisted diagnostic system demonstrated high diagnostic performance: pooled sensitivity = 0.89 (95% CI: 0.87–0.91), specificity = 0.84 (0.80–0.88), positive likelihood ratio (PLR) = 5.60 (4.40–7.20), negative likelihood ratio (NLR) = 0.13 (0.10–0.16), DOR = 43.94 (30.11–64.14), and SROC-AUC = 0.93 (0.91–0.95). The threshold effect analysis (Spearman correlation = -0.18, P > 0.05) indicated no significant heterogeneity. The diagnostic accuracy is higher in Asian countries, in prospective and multicenter designs, with external validation sets, without cross-validation, with deep learning, and in postoperative patient subgroups. Additionally, improved performance was observed in cohorts with smaller nodule diameters (<20 mm), higher malignancy rates, older patient age (≥50 years), and higher female proportions, though heterogeneity remained significant. Univariate and multivariate meta-regression analyses identified AI type, malignancy rate of nodules as significant sources of heterogeneity. Notably, the EDLC-TN model showed the highest diagnostic accuracy.

**Conclusion:**

AI-assisted diagnostic techniques demonstrate significant potentialin thyroid nodule evaluation, with the EDLC-TN model showing particularly high clinical utility. Optimal diagnostic performance was observed for nodules <20 mm in diameter and in patients aged ≥50 years.

**Systematic review registration:**

https://www.crd.york.ac.uk/PROSPERO/view/CRD42024581421, identifier CRD42024581421.

## 1 Introduction

Thyroid nodules are localized, abnormal hyperplastic masses in the thyroid tissue, which are common and frequently occurring diseases. They mainly include nodular goiter, neoplastic nodules, cystic lesions, and inflammatory nodules. With the advantages of being economical and noninvasive, ultrasonography has been widely used for the initial diagnosis and follow-up of thyroid nodules and remains the diagnostic tool of choice in clinical practice (1, 2). The most important thing in the diagnosis and treatment of thyroid nodules is to differentiate between their benign and malignant nature. With the wide application of high-resolution ultrasound technology and the increase in people’s health awareness, the detection rate of thyroid nodules is increasing year by year. However, the traditional ultrasound diagnostic mode is highly dependent on the experienced judgment of radiologists, which has inherent defects such as strong subjectivity and limited diagnostic efficiency (3). In the last 5 years, the rapid development of ultrasound-based artificial intelligence diagnostic systems has brought new methods for the diagnosis of thyroid nodules. This artificial intelligence smart diagnostic system has high accuracy and stability. Practical applications have shown that this auxiliary diagnostic system can effectively reduce the overuse of fine-needle aspiration biopsy (FNA) due to subjective judgment errors and unnecessary surgical operations while significantly shortening the waiting time for patients’ diagnosis and treatment (4, 5). In addition, the technique has good diagnostic accuracy in thyroid nodule diagnosis. Currently, intelligent assistive systems for thyroid nodule diagnosis include various methods such as random forest (RF), machine learning (ML), and convolutional neural network (CNN). However, there are significant differences in diagnostic efficacy among different technical routes, and their clinical application value still needs to be further systematically evaluated.

Existing studies have shown that applying this technology to the clinical diagnosis of thyroid nodules can achieve high accuracy. It is worth noting that due to the relatively short application time of this technology, there is still some controversy in the academic community, and the clinical results vary. For example, the results of the diagnostic model used by WeiX’s team showed a high specificity of 94%, while the results of the diagnostic model used by BudaM’s team showed a specificity of only 52%, which is a significant difference (6). Based on the data from the studies we included, we found a large gap in specificity between the different diagnostic models. Therefore, this study intends to use meta-analysis methods to systematically evaluate the overall performance of intelligent auxiliary diagnosis models based on ultrasound images in the diagnosis of thyroid nodules, to screen out the model with the best diagnostic performance, and to provide a scientific basis for the clinical practice of thyroid cancer.

## 2 Methods

### 2.1 Search strategy and selection criteria

**Figure 1:**
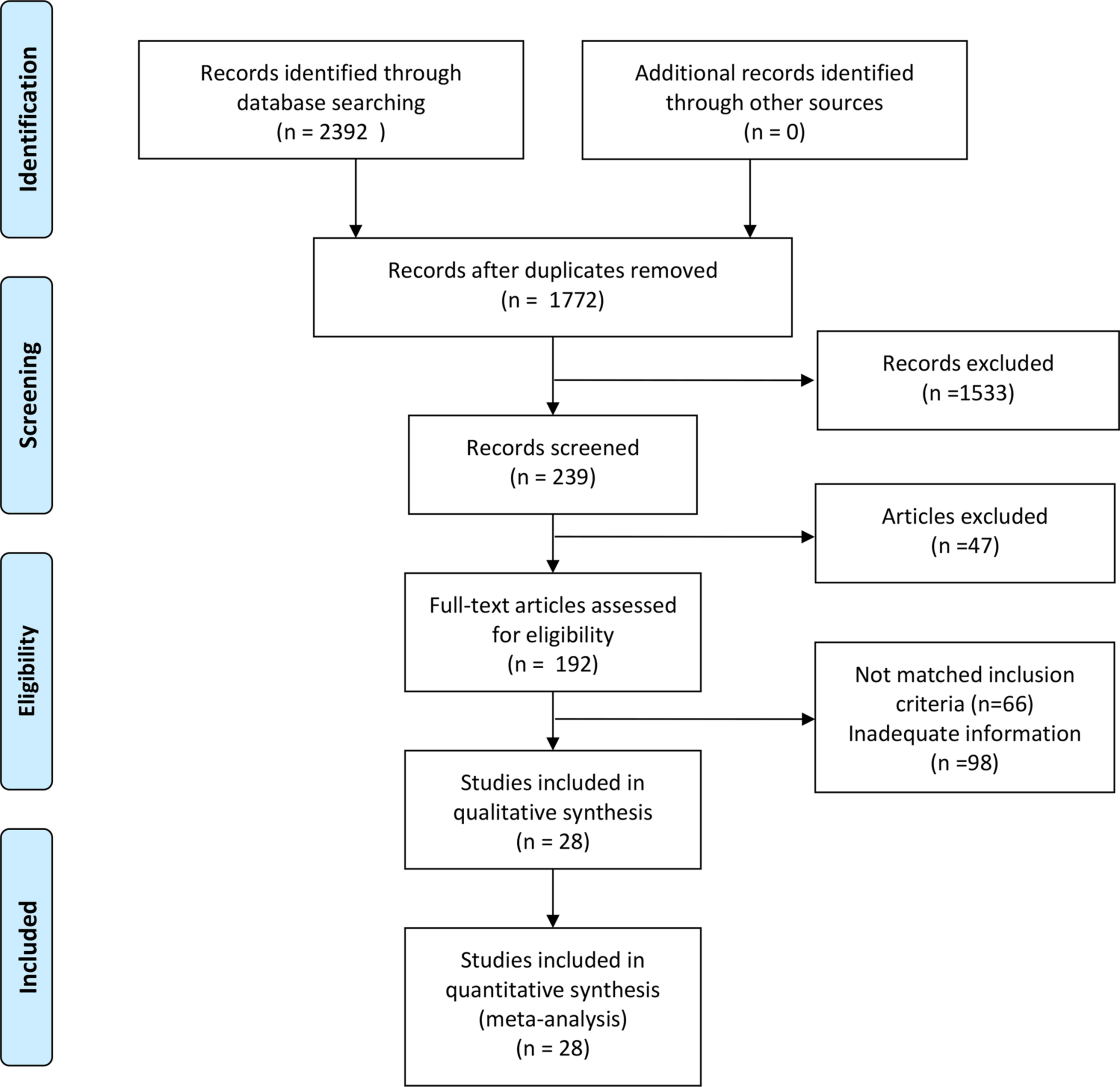
PRISMA diagram for the systematic review.

This study was conducted in strict adherence to the Preferred Reporting Items for Systematic Reviews and Meta-Analyses (PRISMA) guidelines, and the systematic review was registered on PROSPERO at https://www.crd.york.ac.uk/PROSPERO/view/CRD42024581421. A total of 2,392 articles were identified through a literature search across three databases (PubMed: 1,558; Web of Science: 800; Cochrane Library: 34; database inception to August 31, 2024). After removing 620 duplicate records, an additional screening of titles and abstracts excluded studies that did not meet the inclusion criteria, such as those lacking data (n = 26), being irrelevant (n = 1533), conference proceedings (n = 6), or letters (n = 15). This resulted in 192 articles eligible for full-text evaluation. Following a detailed assessment of the complete texts, further exclusions were made due to the unavailability of full texts (n = 1), incomplete data (n = 97), or irrelevance (n = 66). Ultimately, 28 studies were included in the analysis (6–33). We searched using keywords and medical subject terms (MeSH terms): (Artificial Intelligence OR Machine Learning OR Convolutional Neural Network) AND (Thyroid Nodule OR Thyroid Cancer OR Thyroid Neoplasms). Specific search formulas are provided in the Appendix. Two researchers independently performed the literature selection process (**Figure 1**), with discrepancies resolved through discussions during a four-person consensus meeting.

Inclusion criteria: (1) All patients were studied as patients with thyroid nodules, and all underwent ultrasonography; (2) Fine-needle aspiration (FNA) or surgical biopsy was used as the “gold standard” for diagnosing benign and malignant thyroid nodules; (3) Data were complete, and four-quadrant contingency tables could be extracted; (4) Articles generally adhered to the STARD (Standards for Reporting of Diagnostic Accuracy) statement for diagnostic accuracy studies in thyroid cancer research, and (5) At least one artificial intelligence (AI) model was included. Exclusion criteria: (1) non-original research articles, including reviews, meta-analyses, conference proceedings, editorials, letters, and case reports; (2) unavailability of full text; (3) non-English-language publications; and (4) studies with overlapping study populations.

### 2.2 Data extraction

We arranged for two researchers to extract data from each original study and cross-check it independently. Any discrepancies were resolved through discussions within the research team. Specific information extracted from each original study included: first author, year of publication, country, study design, data source, number of cases, study center, malignancy rate of thyroid nodules, number of ultrasound images, reference standard, mean age, proportion of females, mean nodule diameter (in mm), type of artificial intelligence (AI) model, validation set type, depth of study, number of nodules, subgroup status, and diagnostic performance metrics (such us true positives [TP], false positives [FP], false negatives [FN], true negatives [TN]). In this study, for data selection, all external validation sets were used for data extraction and meta-analysis. When multiple external validation sets were available for evaluating AI-assisted diagnostic performance, only the largest cohort was selected for analysis. For nodule attributes, we focused on characteristic data of malignant nodules, including their diameter size, population age, and percentage of females (**Tables 1**–**3**).

**Table 1:** Study characteristics.

Author and year	Country	Data source	No. of patients	Center study	Malignant of all nodules (%)	No. of US images
BudaM2019 (6)	USA	2006.8-2010.5 Duke University School of Medicine	1439	Single	142/1377 (10.3%)	1631
ChenC2024 (7)	China	2019.10-2022.10 Zhejiang,Taizhou Cancer Hospital, Shenzhen People’s Hospital, Shanghai Tenth People’s Hospital	6579	multicenter	2611/6784 (38.5%)	11201
ChenD2020 (8)	China	2011.1-2016.4 Affiliated Hospital of Kunming Medical University	1480	Single	1211/1558 (77.7%)	1558
ChenJH2024 (9)	China	2019.9-2023.2 Shengjing Hospital	300	Single	158/238 (66.4%)	313
ChenYF2022 (10)	China	2018.1-2019.12 Guangdong First hospital 2019.1-2019.12 Guangdong second hospital	636	multicenter	499/1588 (31.4%)	1588
JinZ2022 (11)	China	2013.1-2018.12,2016.5-2019.11 The First Affiliated Hospital of Jinan University,Guangdong Provincial People’s Hospital	3613	multicenter	1085/3965 (27.4%)	3965
KimYJ2022 (12)	Korea	2010.1-2020.3 Mary’s Hospital 2020.1-2020.12 Yeouido St.Mary’s Hospital	7577	multicenter	3194/15409 (20.7%)	15409
KohJ2020 (13)	Korea	2004.1-2019.12 Severance and Kyung Hee Hospital, Samsung and CHA Bundang Medical Center	15375	multicenter	8453/15375 (54.9%)	15375
KoSY2019 (14)	Korea	2012.5-2015.2 Jeju National University Hospital	1576	Single	396/589 (67.2%)	589
LaiM2023 (15)	China	2019.1-2022.9 Zhejiang Cancer Hospital	1242	Single	647/1735 (37.3%)	1735
LiL2024 (16)	China	2020.8-2022.2,2022.3-2022.8 First affiliated hospital of Nanjing medical university	748	Single	381/748 (50.9%)	748
LiX2019 (17)	China	2012.1-2018.3 Tianjin Cancer,Traditional Chinese and Western Medicine Hospital,Jilin,Weihai Municipal Hospital	45644	multicenter	17627/42952 (41%)	396998
NairG2024 (18)	USA	2017.4-2018.5 Stanford dataset,2018.1-2023.12 private practice	277	multicenter	40/314 (12.7%)	314
ParkVY2019 (19)	Korea	2016.6-2017.2 Yonsei University College of Medicine	286	Single	156/286 (54.5%)	4919
SunC2020 (20)	China	2016.6-2016.12 Peking Union Medical College,Beijing Tiantan Hospital	1037	multicenter	651/1037 (62.8%)	1037
VelascoPF2024 (21)	Spain	2021.6-2022.12 Hospital Clínico Universitario Valladolid	172	Single	19/172 (11.1%)	398
WangL2019 (22)	China	2018.1-2018.2 Affiliated Hospital of Qingdao University	276	Single	2557/5007 (51.1%)	5007
WeiX2020 (23)	China	2015.1-2017.12 Tianjin Cancer Institute,Jilin,Cangzhou Chinese and Western Medicine,Peking BinHai Hospital	26541	multicenter	15255/25509 (59.8%)	26541
WuGG2021 (24)	China	2017.6-2019.4 Tongji,Xiangya Hospital	2974	multicenter	1820/5123 (35.5%)	5123
YaoJC2023 (25)	China	2006.5-2022.4 Zhejiang,Taizhou Cancer Hospital,Hangzhou First and Zhejiang People’s Hospital,Sun Yatsen Cancer Center	1690	multicenter	903/1690 (53.4%)	7566
ZhangB2019 (26)	China	2011.4-2016.6 Affiliated Hospital of Jinan University, Guangdong General Hospital	2064	multicenter	750/2064 (36.3%)	2064
ZhaoCK2021 (27)	China	2019.2-2019.4 Ma’anshan People’s Hospital, 2017.9-2019.1 Shanghai Tenth People’s Hospital	822	multicenter	301/849 (35.5%)	849
ZhengYX2024 (28)	China	2009.1-2023.2 Zhejiang Cancer and Zhejiang Provincial People’s Hospital	780	multicenter	257/780 (32.9%)	780
ZhouH2020 (29)	China	2017.1-2018.3 HwaMei Hospital	2284	Single	672/1734 (38.8%)	1734
ZhouTH2024 (30)	China	2017.7-2020.8,2020.9-2021.12 Hangzhou First People’s Hospital,Yantai Yuhuangding and Zhongshan Hospital	637	multicenter	443/637 (69.5%)	1903
ZhuJL2021 (31)	China	2015.1-2017.6 Tianjin Cancer Hospital,2017.8-2017.12 Tianjin Fifth Hospital 2015.1-2017.4 BinHai Hospital	6687	multicenter	10368/18733 (55.3%)	18733
ZhuLC2013 (32)	China	2010.1-2012.12 Affiliated Hospital of Wenzhou Medical College	618	Single	425/689 (61.7%)	689
ZhuYC2022 (33)	China	2021.1-2021.7 Pudong New Area People’s Hospital	674	Single	356/712 (50%)	712

**Table 2:** Study characteristics and population statistics according to malignant nodule class (test group nodules).

Author and year	Design	Reference standard	No.of malignant nodules	Mean size ± SD (mm)	Mean age ± SD (years)	F% (female)
BudaM2019 (6)	Retrospective	FNA,US	15 (15.2%)	27.0 ± 13.0	52.3 ± 14.0	NA
ChenC2024 (7)	Retrospective	FNA,US	504 (38.8%)	11.3 ± 7.2	47.3 ± 12.8	703 (55.7%)
ChenD2020 (8)	Retrospective	SP,US	1211 (77.7%)	NA	43.1 ± 11.4	1178 (79.6%)
ChenJH2024 (9)	Retrospective	SP,FNA,US	21 (67.7%)	10.2 ± 7.83	45.52 ± 11.47	23 (74.2%)
ChenYF2022 (10)	Retrospective	SP,US	66 (27%)	24.0 ± 11.0	46.0 ± 13.0	146 (78%)
JinZ2022 (11)	Retrospective	SP,FNA,US	235 (27.2%)	29.3 ± 11.7	47.5 ± 13.0	578 (75.6%)
KimYJ2022 (12)	Retrospective	FNA,US	25 (42%)	8.96 ± 6.13	52.0 ± 15.0	45 (76%)
KohJ2020 (13)	Retrospective	SP,US	538 (68.9%)	23.6 ± 13.4	47.2 ± 12.9	571 (73.1%)
KoSY2019 (14)	Retrospective	SP,US	100 (66.7%)	12.9 ± 2.3	49.7 ± 12.2	127 (84.7%)
LaiM2023 (15)	Retrospective	SP,FNA,US	44 (35.2%)	10.2 ± 2.7	48.95 ± 3.69	100 (80%)
LiL2024 (16)	Retrospective	SP,FNA,US	80 (50%)	NA	NA	NA
LiX2019 (17)	Retrospective	SP,US	542 (38%)	NA	50.0 ± 9.0	1138 (80%)
NairG2024 (18)	Retrospective	SP,FNA,US	23 (18.9%)	21.0 ± 32.0	61.5 ± 22.6	86 (78.2%)
ParkVY2019 (19)	Prospective	SP,FNA,CNB,US	58 (56.9%)	16.49 ± 1.07	45.9 ± 13.0	81 (85.3%)
SunC2020 (20)	Retrospective	SP,FNA,US	422 (76.7%)	10.5 ± 7.3	42.3 ± 10.7	325 (77%)
VelascoPF2024 (21)	Retrospective	FNA,US	19 (11%)	24.0 ± 11.0	52.3 ± 15.3	143 (83.1%)
WangL2019 (22)	Retrospective	SP,US	181 (65.6%)	11.7 ± 8.7	44.3 ± 11.5	143 (79.01%)
WeiX2020 (23)	Retrospective	SP,US	4330 (62.3%)	13.12 ± 11.49	46 (18–84)	8379 (76.2%)
WuGG2021 (24)	Retrospective	SP,US	509 (44.4%)	15.0 ± 10.0	45.54 ± 11.82	1059 (66.5%)
YaoJC2023 (25)	Retrospective	SP,FNA,US	112 (62.9%)	14.9 ± 7.3	50.0 ± 6.6	131 (73.4%)
ZhangB2019 (26)	Retrospective	SP,US	750 (36.3%)	15.0 ± 10.0	45.25 ± 13.49	1337 (64.8%)
ZhaoCK2021 (27)	Retrospective	FNA,US	31 (30.4%)	18.0 ± 8.43	46.97 ± 10.2	23 (29.9%)
ZhengYX2024 (28)	Retrospective	SP,US	57 (26.1%)	31.0 ± 4.0	51.8 ± 4.5	163 (74.8%)
ZhouH2020 (29)	Retrospective	SP,FNA,US	428 (63.7%)	20.0 ± 10.0	48.6 ± 12.4	833 (64.3%)
ZhouTH2024 (30)	Prospective	SP,FNA,US	225 (77%)	9.81 ± 6.41	44.46 ± 12.02	217 (75%)
ZhuJL2021 (31)	Retrospective	SP,US	530 (51.36%)	NA	52.01 ± 11.83	197 (75.48%)
ZhuLC2013 (32)	Retrospective	SP,FNA,US	425 (61.7%)	13.3 ± 6.5	47.46 ± 11.1	315 (74.1%)
ZhuYC2022 (33)	Retrospective	SP,FNA,US	100 (50%)	14.80 ± 8.25	53.56 ± 14.19	381 (77.9%)

SP, surgical pathology; FNA, fine needle aspiration cytology; US, ultrasonography; CNB core needle biopsy; NA, not applicable.

Data in parentheses are percentages mean data are ± standard deviation.

**Table 3:** Data extraction.

Author and year	Type of AI	Validation	DL	Number of nodules	Group	TP	FP	FN	TN
BudaM2019 (6)	CNN(MDCNN)	Internal Validation (10-fold cross-validation)	Y	1377	1278Training Group+ 99Validation Group	13	40	2	44
ChenC2024 (7)	DCNN(Inception-ResNet)	External Validation	Y	7279	4530Training Group+ 648,1263,138Validation Group	117	2	13	6
ChenD2020 (8)	LLR(Logistic)	Internal Validation (10-fold cross-validation)	N	1370	822Training Group+ 274,274Validation Group	172	17	40	45
ChenJH2024 (9)	RF(2D-US and CEUS)	Internal Validation (10-fold cross-validation)	N	313	282Training Group+ 31Validation Group	17	3	2	9
ChenYF2022 (10)	MTI-RADS (InceptionResNetV2, DCNN)	Internal Validation (5-fold cross-validation)	Y	1588	1345Training Group+ 243Validation Group	55	23	11	154
JinZ2022 (11)	RF(Thy-Wise)	External Validation (10-fold cross-validation)	N	3965	2168Training Group+ 930,867Validation Group	214	229	21	403
KimYJ2022 (12)	DL(VGG16 DCNN)	External Validation	Y	15409	14809Training Group+ 432,168Validation Group	105	87	9	231
KohJ2020 (13)	CNN(DCNN)	External Validation	Y	15375	13560Training Group+ 634,781,200,200Validation Group	130	8	25	37
KoSY2019 (14)	CNN(DCNN)	Internal Validation (3-fold cross-validation)	Y	589	439Training Group+ 150Validation Group	91	9	9	41
LaiM2023 (15)	DCNN(ResNet50)	External Validation (5-fold cross-validation)	Y	1242	894Training Group+ 223,125Validation Group	90	6	15	14
LiL2024 (16)	PLS-DA	External Validation (cross-validation)	N	748	471Training Group+ 117,160Validation Group	100	4	9	47
LiX2019 (17)	DCNN (ResNet50, Darknet19)	External Validation	Y	45644	42952Training Group+ 1118,154,1420Validation Group	461	113	82	764
NairG2024 (18)	AIBx V2(DCNN, ResNet 34)	External Validation	Y	314	192Training Group+ 122Validation Group	20	5	2	95
ParkVY2019 (19)	DCAD(FCN, DCNN)	External Validation	Y	5205	4919Training Group+ 184,102Validation Group	82	6	8	88
SunC2020 (20)	DCNN(VGG-F)	Internal Validation (cross-validation)	Y	1587	1037Training Group+ 550Validation Group	385	25	14	126
VelascoPF2024 (21)	Koios DS(AI-based DSS)	Internal Validation (cross-validation)	Y	172	172Training Group+ 172Validation Group	13	71	2	86
WangL2019 (22)	CAD(YOLOv2NN, DCNN)	Internal Validation (cross-validation)	Y	276	276Training Group+ 276Validation Group	126	14	13	123
WeiX2020 (23)	EDLC-TN (DCNN, DenseNet)	External Validation	Y	25509	17859Training Group+ 1000,6650Validation Group	346	38	25	591
WuGG2021 (24)	DCNN(ResNet50)	External Validation	Y	2295	1289,793Training Group+ 213Validation Group	79	26	21	87
YaoJC2023 (25)	ST	External Validation (10-fold cross-validation)	Y	1690	1349Training Group+ 163,178Validation Group	55	7	11	105
ZhangB2019 (26)	RF	Internal Validation (10-fold cross-validation)	N	2064	1238Training Group+ 826Validation Group	83	116	11	616
ZhaoCK2021 (27)	ML	External Validation	N	849	520Training Group+ 223,106Validation Group	30	16	3	57
ZhengYX2024 (28)	XGBoost	Internal Validation (5-fold cross-validation)	N	780	562Training Group+ 218Validation Group	52	14	4	148
ZhouH2020 (29)	DLRT(DCNN)	External Validation (cross-validation)	Y	1750	1645Training Group+ 105Validation Group	50	8	6	41
ZhouTH2024 (30)	AI-SONIC™Thyroid (DCNN)	External Validation	Y	637	346Training Group+ 291Validation Group	213	8	12	58
ZhuJL2021 (31)	DCNN (BETNET)	Internal Validation	Y	18733	16401Training Group+ 1000,300,1032Validation Group	725	39	51	217
ZhuLC2013 (32)	ANN	Internal Validation (cross-validation)	Y	689	464Training Group+ 225Validation Group	124	14	24	63
ZhuYC2022 (33)	ANN (TDUS-Net)	External Validation(10-fold cross-validation)	Y	712	500Training Group+ 200Validation Group	78	5	22	95

Y, yes; N, NO; TP, true positive; FP, false positive; FN, false negative; TN, true negative; DL, deep learning; ANN, artificial neural network; CNN, convolutional neural network; MDCNN, multitask deep convolutional neural network; DCNN, deep convolutional neural network; ResNet­50,ResNet model18 with 50 layers; Darknet­19,Darknet model 19 with 19 layers; DCAD, deep learning-based US CAD system; FCN, Fully Convolutional Network; CAD, Computer-aided diagnosis systems;YOLOv2NN, YOLOv2 neural network; RF, random forest; LLR, LASSO, logistic regression; LASSO, the least absolute shrinkage and selection operator; SVM, support vector machine; RBF, radial basis function; EDLC-TN, ensemble deep learning classification model for thyroid nodules; DLRT, deep learning Radiomics of thyroid; ML, Machine learning (ML-assisted US visual approach);BETNET, the Brief Efficient Thyroid Network, based on the Visual Geometry Group-19 (VGG-19) model; MTI-RADS, model based on American College of Radiology Thyroid Imaging Reporting and Data System; Thy-Wise, based on a SHapley Additive explanation algorithm; SHAP, SHapley Additive explanation; AIBx V2, AIBx version2; VGG, very deep convolutional networks for large-scale image recognition; TDUS-Net, TI-RADS scores US network mode; ST, swin-transformer; Inception-ResNet, a middle-size model using both depth-wise convolution and residual connected layers; PLS-DA, partial least-squares discriminant analysis; Koios DS(AI-based DSS), artificial intelligence based decision support system); XGBoost, extreme Gradient Boosting; AI-SONIC™Thyroid, AI-assisted diagnostic system(version 5.3.0.2).

### 2.3 Quality assessment

**Figure 2:**
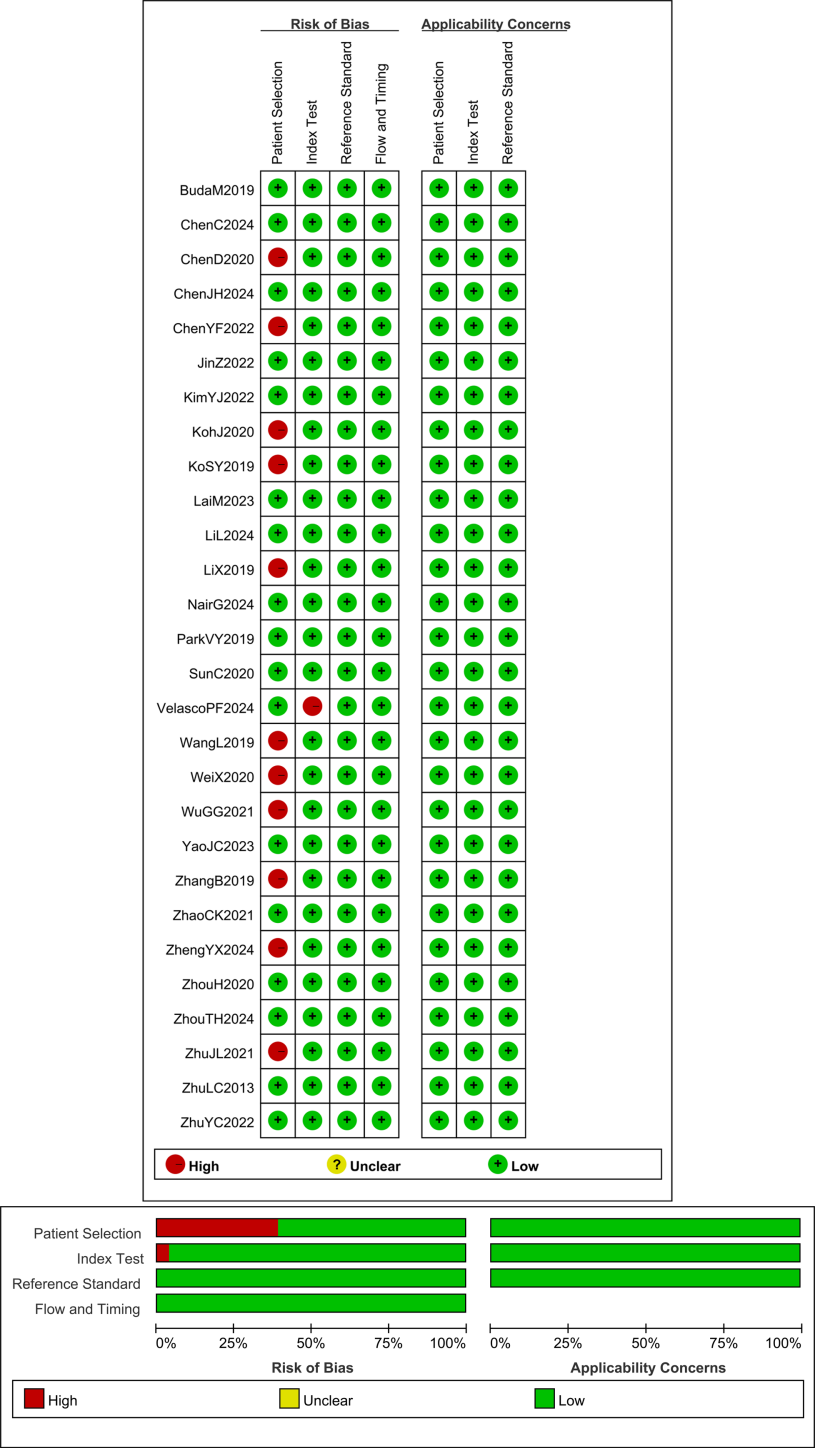
Quality evaluation outcome for each study and summary of quality assessment.

The methodological quality of the included studies was assessed using the Quality Assessment of Diagnostic Accuracy Studies-2 (QUADAS-2) tool, which evaluates the risk of bias and applicability concerns across four domains for the 28 articles. Each risk shift associated with a question is evaluated as “Yes,” “No,” or “Uncertain,” and as “High,” “Low,” or “Uncertain” in terms of its applicability. This assessment was independently completed by two researchers. Any differences that emerged were resolved by the research members through group discussions. Based on the evaluation results, the QUADAS-2 scale was completed, and the quality assessment was subsequently performed using Review Manager 5.4 (**Figure 2**).

### 2.4 Statistical analysis

We used Meta-Disc 1.4, Review Manager 5.4, R 4.4.2, and Stata 17.0 software for statistical analysis. Diagnostic efficacy was evaluated by constructing and summarizing ROC (SROC) curves by combining sensitivity, specificity, diagnostic advantage ratio (DOR), and AUC at 95% CI. The Deeks’ Funnel Plot Asymmetry Test was then used to assess publication bias. The Spearman correlation coefficient was also used to evaluate the threshold effect between studies. For heterogeneity evaluation, we used Cochran’s Q-test and I-squared (I^2^) statistic. Univariate and multivariate meta-regression analyses were employed to identify the source of heterogeneity, and subgroup analyses were conducted. Variables that produced heterogeneity were analyzed, and p-values < 0.05 were considered statistically significant.

## 3 Results

### 3.1 Literature search and study characteristics

After a comprehensive literature review and checking, 192 records were finally obtained. Unavailability of full text (1), incomplete data (97), and irrelevant studies (66) were excluded. Finally, a total of 28 studies that met the criteria were included in this meta-analysis (**Figure 1**). Of the 28 included studies, all were in English, 21 studies were from China (75%), 4 studies were from Korea (14%), 2 studies were from the United States (7%), and 1 study was from Spain (4%).25 studies were from Asian countries (89%), and 3 studies were from Western countries (11%).2 studies were prospective studies (7%), and 26 studies were retrospective (93%). In terms of diagnostic models, these included CNN 17 studies (61%), ANN 2 studies (7%), RF 3 studies (11%), and Mixed Model Group 6 studies (21%). Twelve studies employed a single-center design (43%), while 16 studies utilized a multicenter design (57%). 16 studies used an external validation set (57%), and 12 studies used an internal validation set (43%). Seventeen studies performed cross-validation (61%), while 11 studies did not (39%). 21 studies employed deep learning (75%), and 7 studies used non-deep learning (25%). 11 studies selected only cases after thyroid surgery (39%), and 17 studies were FNA cases (61%). Sixteen studies were thyroid nodules with a mean diameter of < 20mm. Nine studies included patients with a mean age of 50 years or older. The malignant rate of thyroid nodules was ≥50% (50%) in 14 studies and < 50% (50%) in the remaining 14 studies (**Tables 4**, **5**). In addition, this meta-analysis included a total of 134,028 patients, 158,161 thyroid nodules, and 529,479 ultrasound images of thyroid nodules (**Tables 1**–**3**).

**Table 4:** The results of subgroup analyses.

Subgroup	No. of models	SEN			P^b^	SPE			P^b^	I2 (95%CI)^c^	P^c^	Pooled DOR	AUC	P^d^
		Pooled SEN	I2%	P^a^		Pooled SPE	I2%	P^a^						
Country														
Asian	25	0.89 (0.87-0.91)	80.16	0.00	0.06	0.85 (0.82-0.88)	92.96	0.00	0.68	34 (0-100)	0.22	48.11 (34.07-67.92)	0.94 (0.91-0.96)	0.73
Western	3	0.88 (0.76-0.95)	0.00	0.74		0.74 (0.39-0.93)	97.04	0.00				21.38 (3.18-143.87)	0.89 (0.86-0.92)	
Design														
Retrospective	26	0.89 (0.87-0.91)	76.82	0.00	0.00	0.83 (0.79-0.87)	94.41	0.00	0.02	44 (0-100)	0.17	40.08 (27.34-58.77)	0.93 (0.90-0.95)	0.84
Prospective	2	0.94 (0.90-0.96)	26.84	0.24		0.91 (0.85-0.95)	37.46	0.21				149.7 (71.95-311.45)	0.97 (0.95-0.98)	
AI algorithms														
CNN	17	0.90 (0.88-0.92)	80.12	0.00	/	0.85 (0.80-0.89)	91.19	0.00	/	/	/	51.33 (31.33-84.09)	0.94 (0.92-0.96)	/
ANN	2	0.81 (0.76-0.86)	24.32	0.25	/	0.90 (0.76-0.96)	87.32	0.00	/	/	/	38.83 (15.83-95.28)	0.88 (0.84-0.90)	/
RF	3	0.90 (0.85-0.93)	22.85	0.27	/	0.75 (0.62-0.85)	97.32	0.00	/	/	/	26.89 (14.54-49.72)	0.91 (0.89-0.94)	/
Mixed	6	0.86 (0.81-0.90)	56.05	0.04	/	0.84 (0.71-0.92)	95.52	0.00	/	/	/	32.51 (11.47-92.11)	0.90 (0.88-0.93)	/
Center study														
Single	12	0.87 (0.83-0.90)	45.52	0.04	0.00	0.82 (0.73-0.89)	93.05	0.00	0.00	28 (0-100)	0.25	30.1 (15.95-56.79)	0.90 (0.88-0.93)	0.81
multicenter	16	0.90 (0.88-0.93)	82.78	0.00		0.85 (0.81-0.89)	95.52	0.00				55.14 (35.43-85.80)	0.94 (0.92-0.96)	
Validation type														
External	16	0.89 (0.86-0.91)	74.75	0.00	0.00	0.87 (0.81-0.91)	95.75	0.00	0.00	24 (0-100)	0.27	51.03 (31.85-81.76)	0.94 (0.91-0.95)	0.87
Internal	12	0.90 (0.86-0.93)	81.45	0.00		0.80 (0.73-0.86)	92.42	0.00				35.38 (18.66-67.11)	0.93 (0.90-0.95)	
Cross-validation														
CV	17	0.89 (0.86-0.91)	74.16	0.00	0.00	0.82 (0.76-0.88)	94.34	0.00	0.00	0 (0-100)	0.43	36.64 (22.92-58.58)	0.93 (0.90-0.95)	0.54
NCV	11	0.90 (0.87-0.93)	82.10	0.00		0.86 (0.81-0.90)	91.04	0.00				58.95 (32.91-105.61)	0.95 (0.92-0.96)	
Deep learning														
DL model	21	0.89 (0.87-0.91)	80.35	0.00	0.00	0.85 (0.80-0.89)	93.41	0.00	0.01	0 (0-100)	0.78	46.52 (29.93-72.31)	0.94 (0.91-0.95)	0.12
NDL model	7	0.89 (0.85-0.92)	79.33	0.00		0.81 (0.72-0.88)	95.13	0.00				36.58 (17.93-74.63)	0.93 (0.90-0.95)	
Reference standard														
SP	11	0.88 (0.85-0.91)	83.50	0.00	0.00	0.86 (0.82-0.89)	83.22	0.00	0.00	0 (0-100)	0.42	47.32 (27.99-80.02)	0.94 (0.91-0.95)	0.75
FNA	17	0.90 (0.87-0.92)	71.44	0.00		0.83 (0.75-0.88)	94.13	0.00				44.33 (26.85-73.19)	0.94 (0.91-0.95)	

SP, Surgical pathology; SEN, sensitivity; SPE, specificity; PLR, positive likelihood ratio; NLR, negative likelihood ratio; AUC, area under the curve; CV, cross-validation; NCV, non-cross-validation; NDL, non-deep learning; DL, deep learning; AI, artificial intelligence; ANN, artificial neural network; CNN, convolutional neural network; RF, random forest; FNA, fine needle aspiration cytology; 95% CI: 95% confidence interval; ^a^P value for heterogeneity within each subgroup; ^b^P value for heterogeneity between subgroups with meta-regression analysis; ^c^P value for joint model estimates; ^c^I2 (95%CI) for heterogeneity between subgroups with meta-regression analysis; ^d^P value for Multivariate Meta-regression.

**Table 5:** Summary estimate of Univariable meta-regression analysis.

Subgroup	No. of models	Sensitivity			P^b^	Specificity			P^b^	I2 (95%CI)^c^	P^c^	Pooled DOR (95% CI)	AUC (95% CI)	P^d^
		SEN (95% CI)	I2	P^a^		SPE (95% CI)	I2	P^a^						
Number of patients														
≥500	23	0.89 (0.87-0.91)	81.05	0.00	0	0.84 (0.79-0.87)	93.8	0.00	0.00	0 (0-100)	0.81	41.78 (28.48-61.27)	0.93 (0.91-0.95)	0.83
<500	5	0.91 (0.85-0.96)	0.00	0.93		0.86 (0.77-0.95)	97.09	0.00				58.16 (17.99-187.96)	0.92 (0.89-0.94)	
Malignant of all nodules (%)														
≥50%	14	0.9 (0.88-0.92)	85.97	0.00	0	0.88 (0.84-0.92)	78.69	0.00	0.00	68 (27-100)	<0.05	67.68 (42.57-107.58)	0.95 (0.93-0.97)	0.04
<50%	14	0.88 (0.85-0.91)	31.98	0.12		0.79 (0.73-0.85)	95.28	0.00				27.40 (18.03-41.63)	0.90 (0.87-0.93)	
AI algorithms														
CNN	17	0.90 (0.88-0.92)	80.12	0.00	0	0.85 (0.80-0.89)	91.19	0.00	0.00	11 (0-100)	0.32	51.33 (31.33-84.09)	0.94 (0.92-0.96)	0.05
Mixed	11	0.87 (0.83-0.90)	66.26	0.00		0.83 (0.75-0.89)	95.42	0.00				33.12 (19.29-56.86)	0.91 (0.88-0.93)	
Number of nodules														
≥500	24	0.89 (0.87-0.91)	80.41	0.00	0	0.84 (0.80-0.88)	93.82	0.00	0.05	0 (0-100)	0.95	43.95 (30.00-64.38)	0.93 (0.91-0.95)	0.57
<500	4	0.90 (0.84-0.97)	0.00	0.90		0.83 (0.72-0.94)	97.13	0.00				44.87 (11.66-172.74)	0.91 (0.89-0.94)	
Population statistics according to malignant nodule class (test nodules)														
No. of malignant nodules														
≥30%	22	0.89 (0.87-0.91)	81.44	0.00	0	0.86 (0.82-0.90)	84.05	0.00	<0.05	7 (0-100)	0.34	49.23 (34.21-70.85)	0.94 (0.91-0.96)	0.30
<30%	6	0.89 (0.84-0.95)	47.31	0.09		0.78 (0.68-0.88)	97.28	0.00				32.34 (13.23-79.02)	0.90 (0.87-0.93)	
Mean size (mm)														
≥20	8	0.88 (0.85-0.91)	48.22	0.06	0	0.80 (0.67-0.89)	96.2	0.00	0.00	97 (95-99)	0.00	29.88 (15.17-58.85)	0.90 (0.87-0.92)	0.38
<20	16	0.90 (0.87-0.92)	78.58	0.00		0.86 (0.81-0.89)	87.34	0.00				53.82 (34.70-83.49)	0.94 (0.92-0.96)	
Mean age														
≥50	9	0.89 (0.85-0.92)	82.16	0.00	0	0.85 (0.74-0.92)	96.4	0.00	0.00	81 (60-100)	0.00	46.85 (25.24-86.96)	0.93 (0.91-0.95)	0.07
<50	18	0.89 (0.87-0.92)	78.66	0.00		0.83 (0.79-0.87)	92.84	0.00				42.26 (27.20-65.67)	0.93 (0.91-0.95)	
F% (Female)														
≥70%	21	0.90 (0.87-0.92)	81.76	0.00	0	0.85 (0.81-0.89)	95.45	0.00	0.04	92 (85-99)	0.00	50.01 (32.64-76.62)	0.94 (0.91-0.96)	0.21
<70%	5	0.87 (0.82-0.91)	47.07	0.11		0.82 (0.78-0.85)	26.58	0.24				31.42 (18.69-52.80)	0.90 (0.87-0.92)	

95% CI, 95% confidence interval.

^a^P value for heterogeneity within each subgroup.

^b^P value for heterogeneity between subgroups with meta-regression analysis.

^c^P value for joint model estimates.

^c^I2(95%CI) for heterogeneity between subgroups with meta-regression analysis.

^d^P value for Multivariate Meta-regression.

### 3.2 Study quality assessment

The QUADAS-2 scale was employed to assess the methodological quality of the 28 articles included in this study. Among these, 11 studies exhibited case selection bias because the cases selected by the original authors were exclusively surgical patients, which may have resulted in a significantly higher risk of thyroid cancer in this group. One study demonstrated index test bias due to using the same data set to train and test the same data set to train and test the AI diagnostic model, comprising a total of 172 nodules. However, since the original authors mitigated overfitting through cross-validation, this study can still be rated as low risk in terms of applicability (21). The results showed that the research quality of the studies included in this meta-analysis was satisfactory (**Figure 2**).

### 3.3 Diagnostic accuracy and heterogeneity evaluation

We analyzed the threshold effect by Meta-Disc, and the results showed that the Spearman correlation coefficient value was -0.18, P>0.05, no statistical significance, the threshold effect caused no heterogeneity, and we could continue to merge the effect sizes of the diagnostic models and meta-analysis of related data.

**Figure 3:**
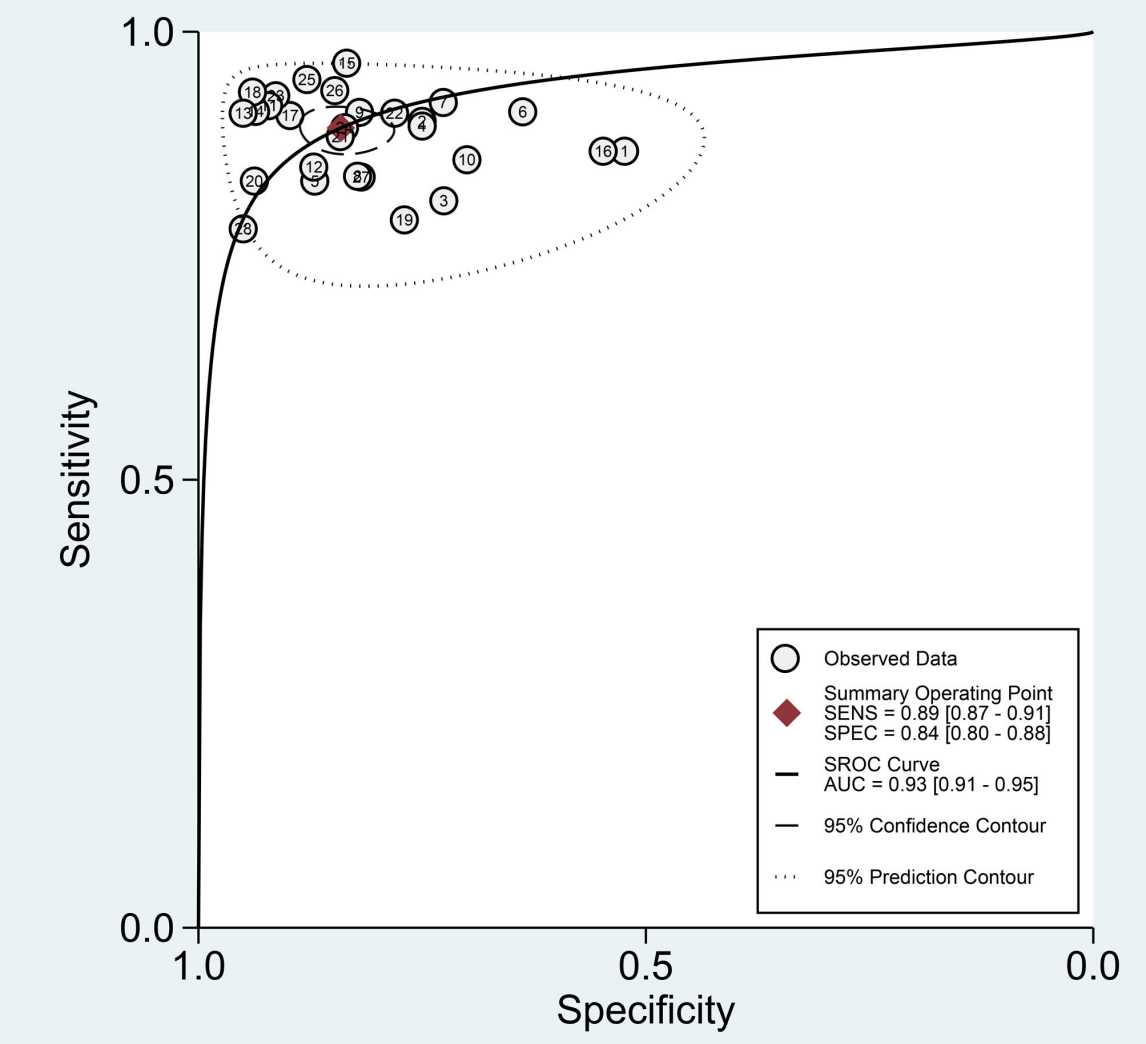
SROC curve for diagnosis of thyroid nodules by AI-aided diagnostic models.

Among the 28 studies included in the meta-analysis, the diagnostic performance of AI-assisted diagnostic technology for thyroid nodules was evaluated. The results indicated that the AI-assisted diagnostic system demonstrated good diagnostic efficacy for distinguishing benign and malignant thyroid nodules: the pooled sensitivity was 0.89 (95% CI: 0.87–0.91); the pooled specificity was 0.84 (95% CI: 0.80–0.88); the positive likelihood ratio (PLR) was 5.60 (95% CI: 4.40–7.20); the negative likelihood ratio (NLR) was 0.13 (95% CI: 0.10–0.16); and the diagnostic odds ratio (DOR) was 43.94 (95% CI: 30.11–64.14) (**Table 6**). Additionally, the area under the curve (AUC) for the summary receiver operating characteristic (SROC) plot was 0.93 (95% CI: 0.91–0.95) (**Figure 3**).

**Table 6:** Meta-analysis results of AI-assisted diagnosis of thyroid nodules.

Statistical measure	Pooled estimate(95%CI)	Cochran’s Q	I2(%)	P value
Pooled sensitivity	0.89 [0.87-0.91]	117.66	77.05	0.00
Pooled specificity	0.84 [0.80-0.88]	476.82	94.34	0.00
Pooled positive likelihood ratio	5.60 [4.40-7.20]	422.45	92.03	0.00
Pooled negative likelihood ratio	0.13 [0.10-0.16]	118.91	77.29	0.00
Pooled diagnostic odd ratio	43.94 [30.11-64.14]	144.27	81.28	0.00

AI, artificial intelligence.

**Figure 4:**
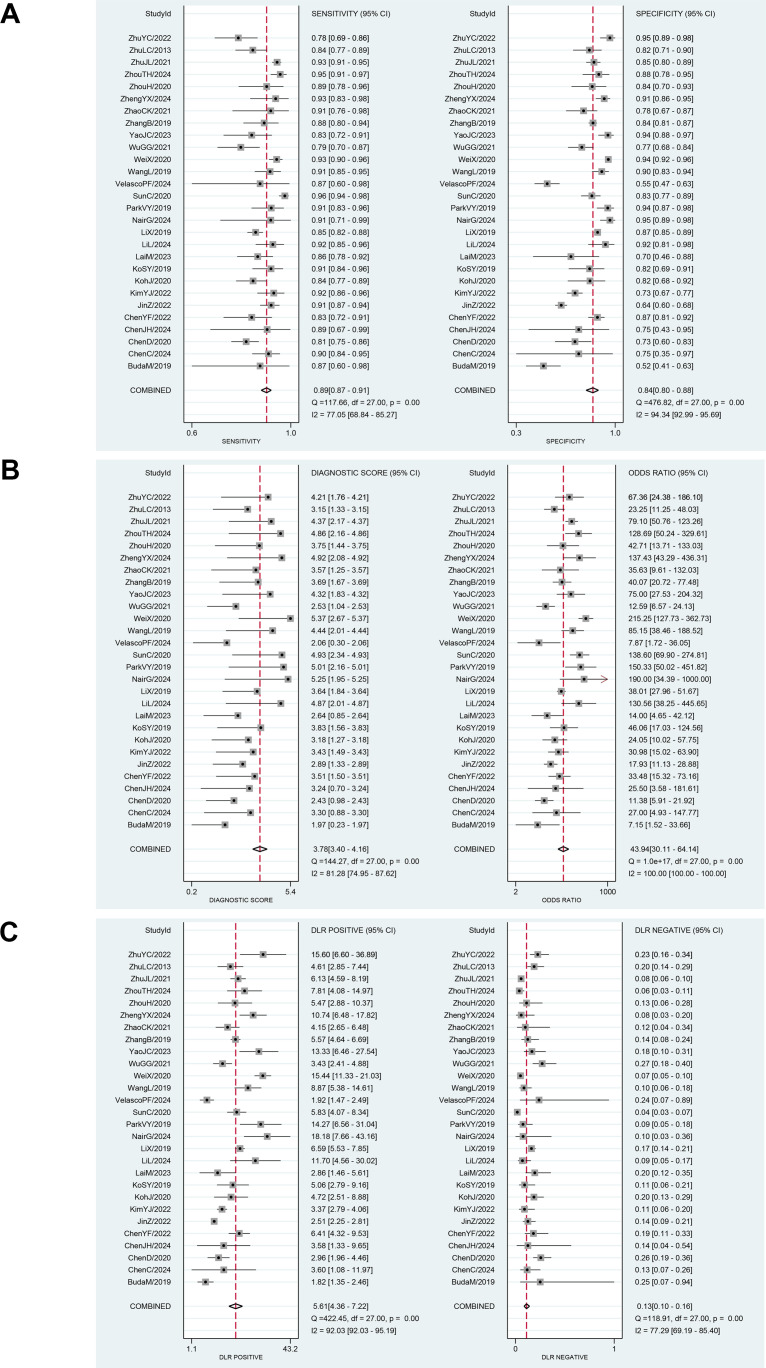
**(A)** Forest plot for sensitivity and specificity after combination. **(B)** Forest plot for diagnostic odds ratio and diagnostic score after combination. **(C)** Forest plot for likelihood ratio after combination (LR+, LR-).

Although these results indicate that AI-assisted diagnostic techniques exhibit good diagnostic efficacy for thyroid nodules, significant heterogeneity was observed in the pooled analysis of sensitivity and specificity. In the meta-analysis, the heterogeneity of sensitivity for AI-assisted diagnostic techniques was I² = 77.05% (95% CI: 68.84%-85.27%), P = 0, while the heterogeneity of specificity was I² = 94.34% (95% CI: 92.99%-95.69%), P = 0. These findings suggest that although the diagnostic sensitivity and specificity of the thyroid AI-assisted diagnostic system are high, there is substantial heterogeneity among studies (I² > 50%, P = 0) (**Figure 4A**). The forest plot of the diagnostic odds ratio (DOR) demonstrated a pooled DOR value of 43.94 and a diagnostic score of 3.78, indicating that the AI diagnostic model has strong diagnostic value and can be applied to evaluate individual clinical cases (**Figure 4B**). In addition, we did forest plots of positive likelihood ratio (PLR) and negative likelihood ratio (NLR), and we found that positive likelihood ratio = 5.61 and negative likelihood ratio = 0.13, which indicates that the AI diagnostic model has a high diagnostic value for thyroid nodules (**Figure 4C**).

**Figure 5:**
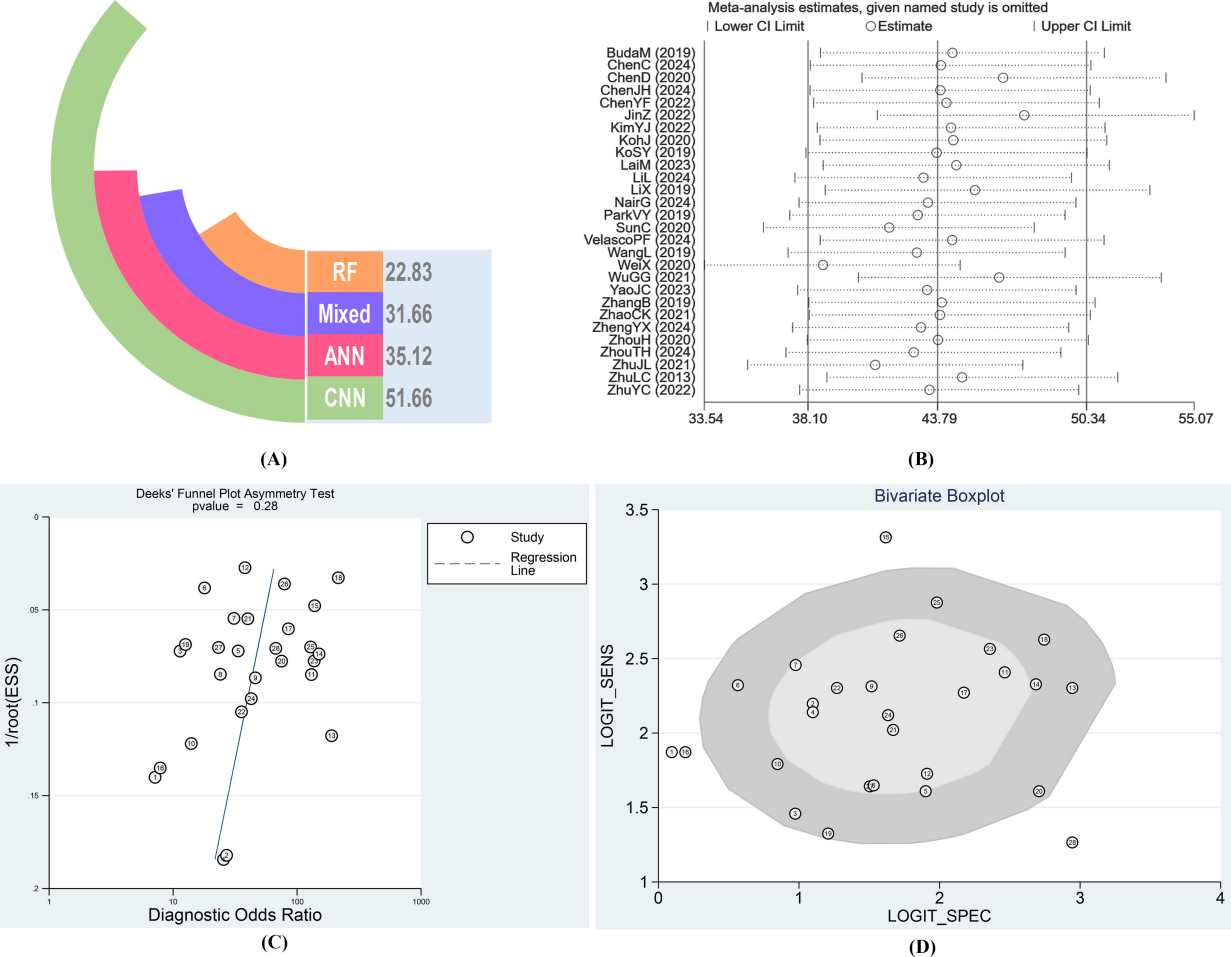
**(A)** Diagnostic Odds Ratio of different AI-aided diagnostic models after combination. **(B)** Result of Sensitivity analysis. **(C)** Results of Deeks’ Funnel Plot of Asymmetry Test for publication bias. **(D)** Bivariate Boxplot for diagnosis of thyroid nodules by AI.

Given the large number of studies included, there may be other factors influencing the overall results. Therefore, we conducted a sensitivity analysis (**Figure 5B**), sequentially excluding individual studies, and did not find any study with apparent heterogeneity. To identify heterogeneity, we also performed a Bivariate Boxplot analysis, which indicated that four articles exhibited significant heterogeneity: 1 (BudaM2019), 15 (SunC2020), 16 (VelascoPF2024), and 28 (ZhuYC2022) (**Figure 5D**). Sensitivity analysis results indicated that the four individual studies did not significantly impact the stability of the results. Therefore, we conducted further sensitivity analysis by simultaneously excluding the four articles identified in the Bivariate Boxplot analysis. After exclusion, the sensitivity I² was 73.47% (95% CI: 62.79%–84.15%), P = 0.00. The specificity I² was 93.36% (95% CI: 91.56%–95.16%), P = 0.00, indicating no significant changes in overall sensitivity and specificity heterogeneity. Thus, the results of the 28 studies included in this analysis demonstrate high stability across studies (**Supplementary Figure S1**). We conducted a publication bias test, with the Deeks’ Funnel Plot of Asymmetry Test yielding a p-value of 0.28, indicating that the studies were generally symmetrical. We also performed an Egger test (p = 0.992), which confirmed the absence of publication bias across the studies (**Figure 5C**). Given that all included studies in this research were of high quality and stability, the AI type of these four articles does not substantially impact the results, and the findings of this study are highly reliable.

After excluding the influence of threshold effects on heterogeneity, we conducted a subgroup univariable meta-regression analysis based on the completeness of the collected data, including (country/region, study design, number of centers, validation type, cross-validation, deep learning, reference standard) to determine the sources of heterogeneity in sensitivity and specificity. We found that six subgroups, excluding region, had statistically significant effects on the heterogeneity of sensitivity and specificity. The results of the joint model showed that no subgroup had a statistically significant impact on the heterogeneity of sensitivity and specificity. The diagnostic performance results of the AI diagnostic model indicated that Asian countries, prospective studies, multicenter studies, studies using external validation sets, non-cross-validation, deep learning models, and the subgroup of patients who underwent thyroid surgery had higher diagnostic accuracy, but also exhibited higher heterogeneity. AI models demonstrated high diagnostic performance in Asian populations, with a sensitivity of 0.89 (0.87–0.91) and a specificity of 0.85 (0.82–0.88). In prospective studies, AI models performed notably well, with an AUC of 0.97 (0.95–0.98). In multicenter studies, the AI diagnostic model demonstrated high sensitivity (0.90, 95% CI 0.88–0.93) and specificity (0.85, 95% CI 0.81–0.89). Additionally, when an external validation set was available, the diagnostic model exhibited high diagnostic performance, with an AUC of 0.94 (95% CI 0.91–0.95). The non-cross-validation group had a higher AUC of 0.95 (0.92–0.96). In the deep learning subgroup, the deep learning group had a high AUC of 0.94 (0.91–0.95). Finally, the subgroup of patients who underwent surgery alone had high diagnostic accuracy, with a pooled DOR of 47.32 (27.99–80.02) (**Table 4**).

**Figure 6:**
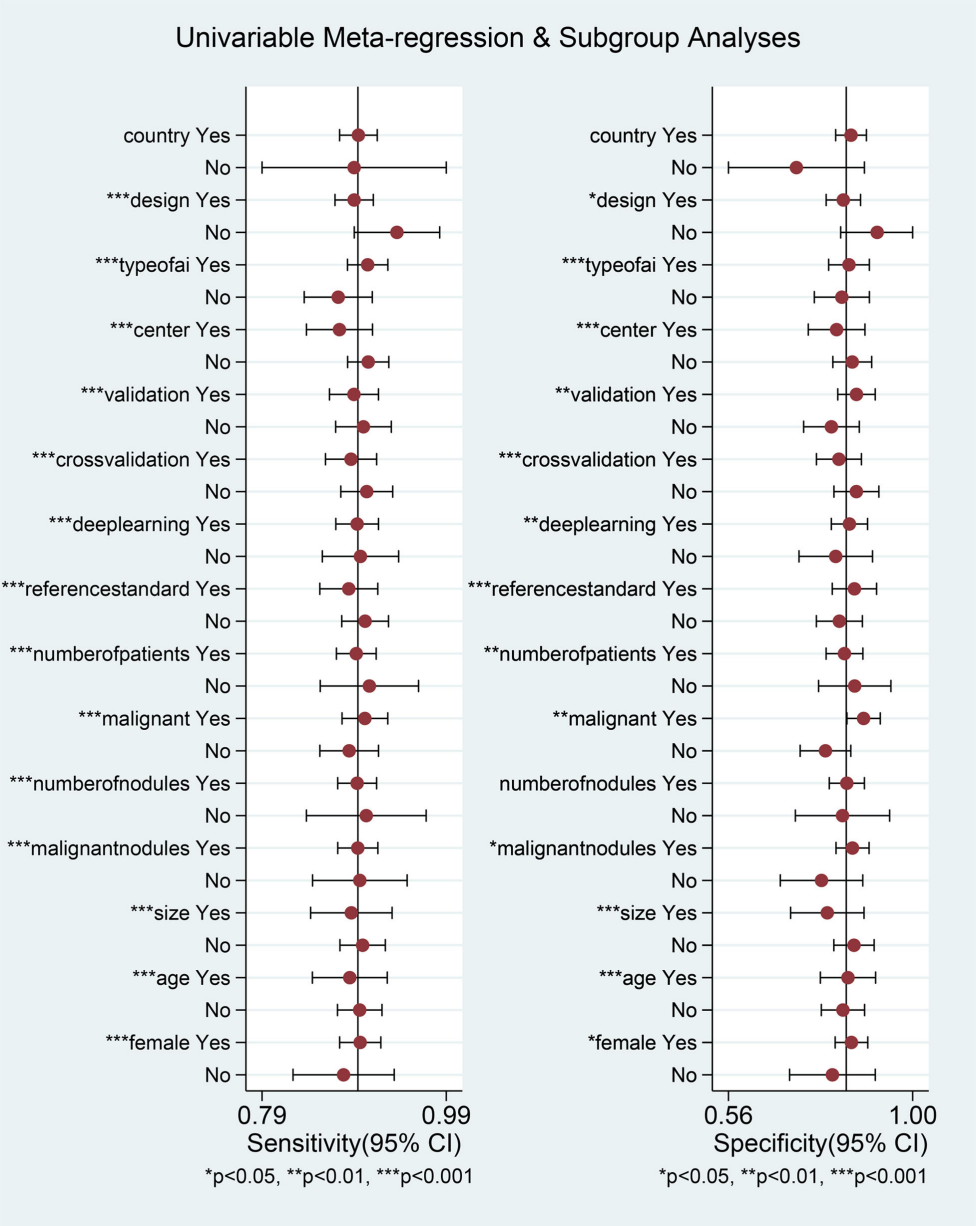
Univariable meta-regression analysis.

We also conducted univariable meta-regression analysis on the remaining eight subgroups (number of patients, overall malignancy rate, AI type, nodule count, nodule malignancy rate, nodule diameter, patient age, and female proportion) using univariable meta-regression analysis. We found that, except for nodule count, which had a statistically significant effect on sensitivity heterogeneity but no statistically significant effect on specificity heterogeneity, the remaining seven subgroups all had statistically significant effects on both sensitivity and specificity heterogeneity (**Figure 6**, **Table 5**). The results of the joint model showed that the four subgroups—thyroid nodule malignancy rate, nodule size, patient age, and female proportion — had statistically significant effects on the heterogeneity of sensitivity and specificity. The results of the AI diagnostic model’s diagnostic performance showed that the diagnostic performance of the AI diagnostic model was stronger when the number of patients and nodules was higher, the nodule malignancy rate was higher, the convolutional neural network (CNN) was used, the nodule diameter was smaller, and the patient age and female proportion were higher. The AI model demonstrated high diagnostic performance in studies with a large sample size (≥500 patients), with an AUC of 0.93 (0.91–0.95). When the thyroid nodule malignancy rate was ≥50%, the AI model exhibited a sensitivity of 0.90 (0.88–0.92) and specificity of 0.88 (0.84–0.92). Among AI model subtypes, the convolutional neural network (CNN) model performed best, with a sensitivity of 0.90 (0.88–0.92) and specificity of 0.85 (0.80–0.89). The more nodules included in the study, the better the diagnostic performance of the AI model, with an AUC of 0.94 (0.91–0.96). The higher the malignancy rate of the nodules, the better the diagnostic performance of the model, with an AUC of 0.94 (0.91–0.96). The AI-assisted diagnostic model demonstrated higher sensitivity (0.90 [0.87–0.92]) and specificity (0.86 [0.81–0.89]) for thyroid nodules with an average diameter <20 mm. For patients with thyroid nodules aged 50 years or older, the AI-assisted diagnostic model has higher diagnostic value, although the difference is not statistically significant. The AI-assisted diagnostic model has higher sensitivity (0.90, 95% CI: 0.87–0.92) and specificity (0.85, 95% CI: 0.81–0.89) in populations with a higher proportion of female patients (**Table 5**). Finally, considering the heterogeneity of the included studies, we conducted a multi-factor meta-regression analysis by including all variables in the model, in addition to the results of the combined model. The results showed that nodule malignancy rate, and AI type had statistically significant heterogeneity in sensitivity and specificity, and were significant sources of heterogeneity (**Tables 4**, **5**).

**Figure 7:**
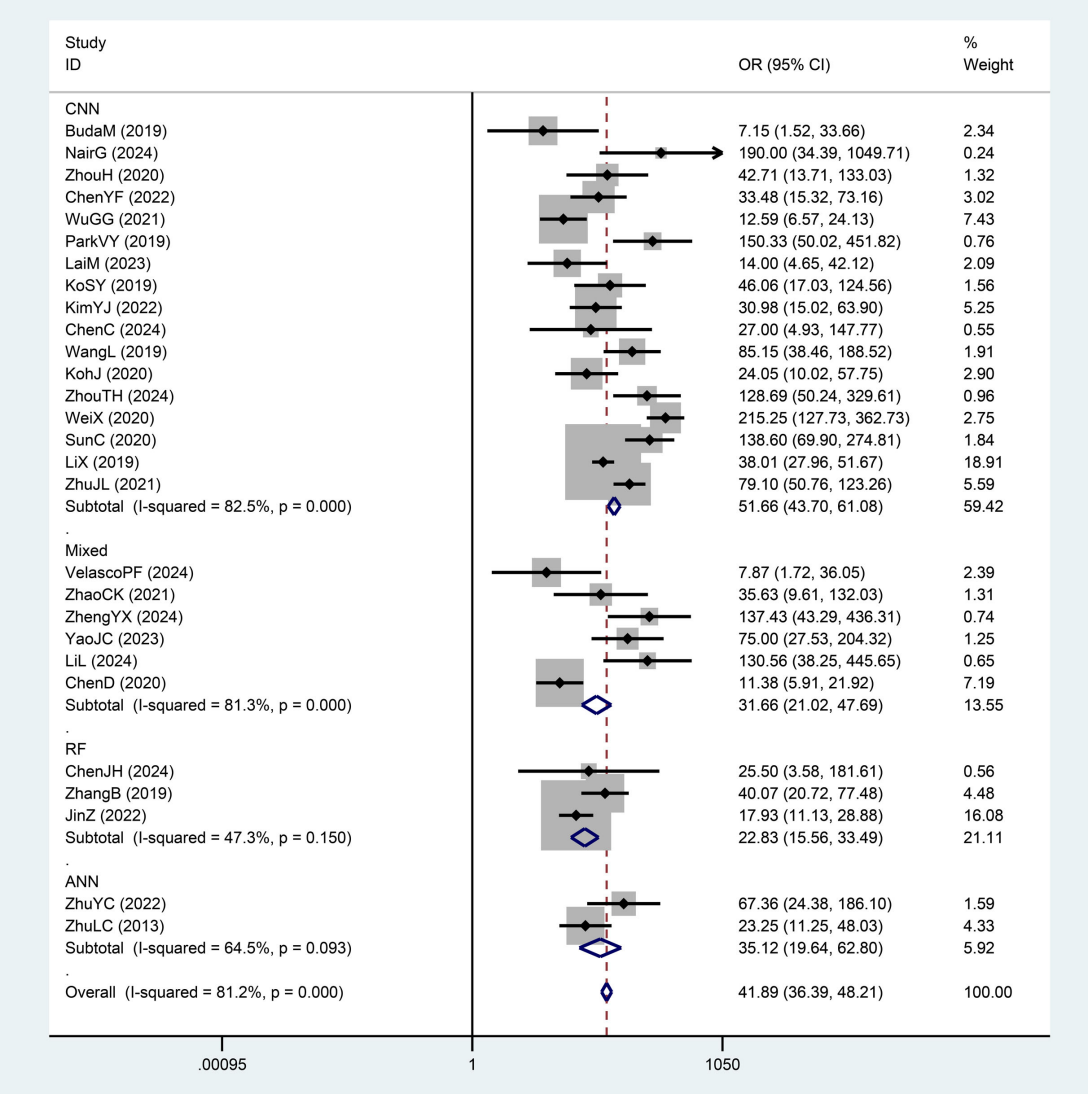
The results of subgroup analyses(AI algorithms).

We divided the AI-assisted diagnostic system into four subgroups: CNN, RF, ANN, and Mixed Model Group. By comparing the DOR and weights of each subgroup, we found significant heterogeneity within the CNN and Mixed Model Group subgroups. The diagnostic odds ratio (DOR) indicates the strength of the association between the diagnostic test results and the disease; a higher value indicates better discriminatory performance. Since the AI diagnostic models differ across subgroups, we compared and analyzed the overall DOR values between different diagnostic model subgroups, finding significant differences between groups. The RF group had the lowest DOR value (22.83), while the CNN group had the highest DOR value (51.66). The AI diagnostic model of the CNN group demonstrated a significantly higher value for distinguishing benign from malignant thyroid nodules compared to the Mixed Model Group, RF, and ANN groups (**Figures 7**, **5A**).

**Figure 8:**
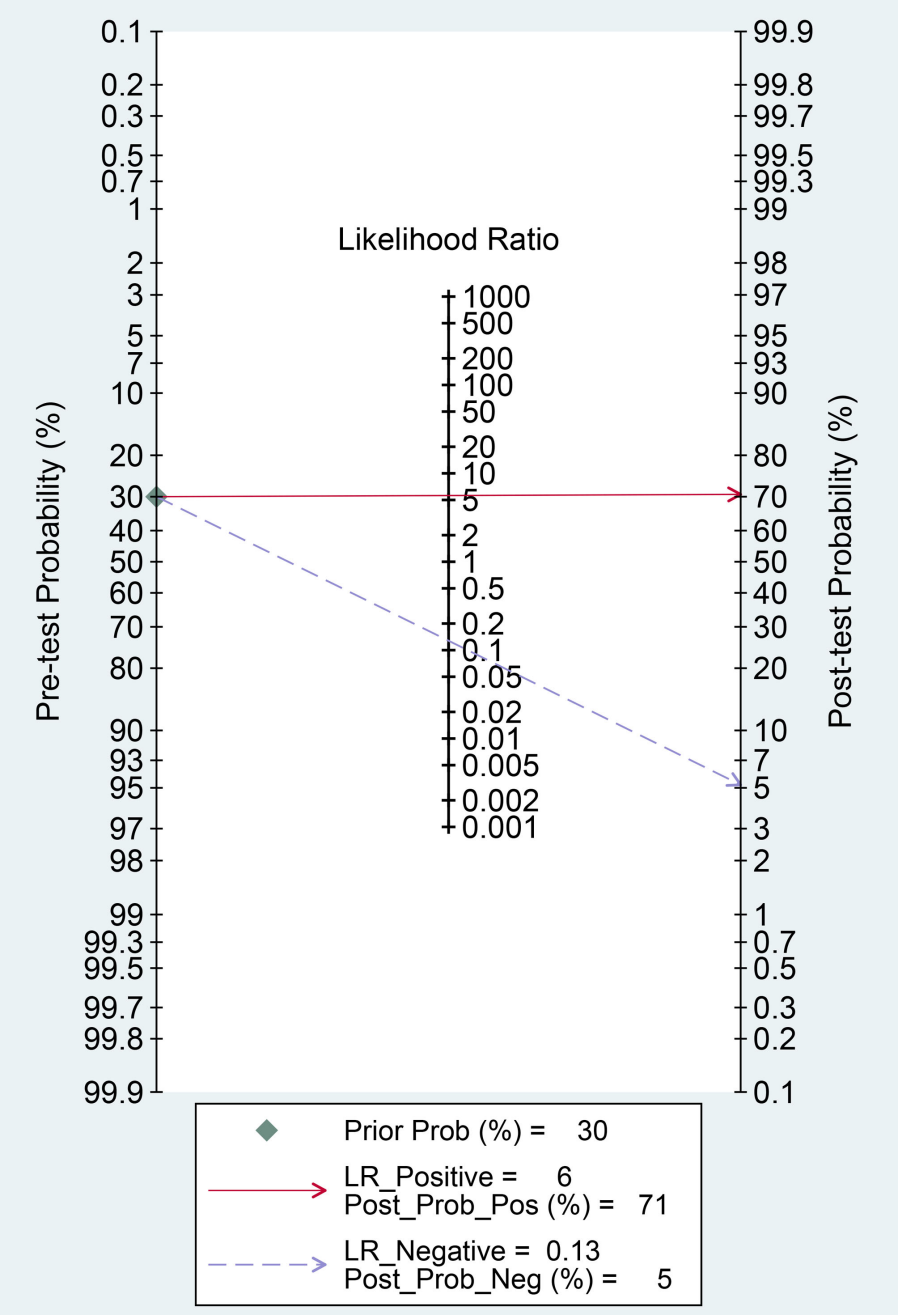
Fagan nomogram: Evaluation of clinical applicability of AI-assisted diagnostic techniques in thyroid nodules diagnosis.

**Figure 9:**
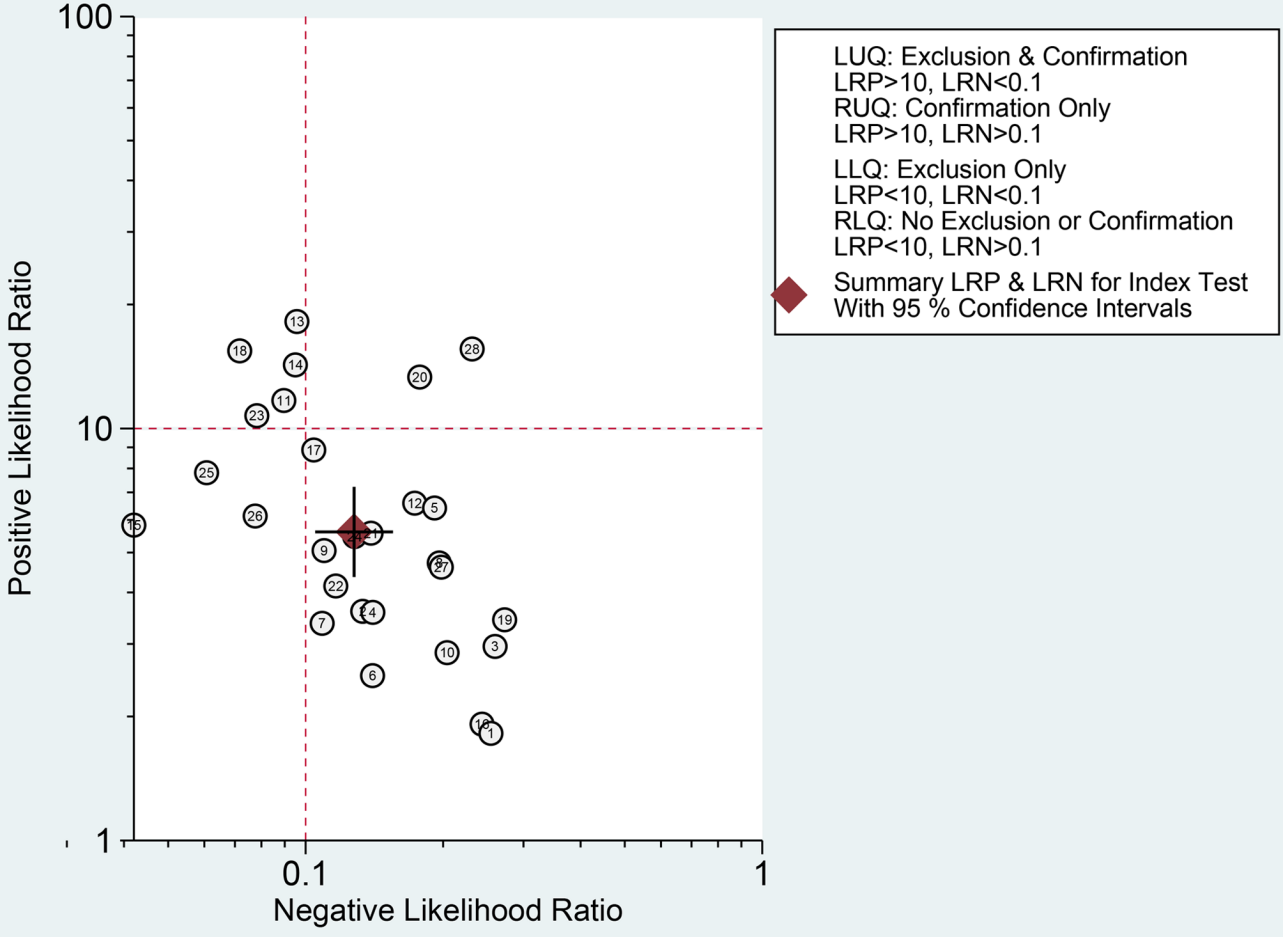
Likelihood ratio scattergram: Evaluation of clinical applicability of AI-assisted diagnostic techniques in thyroid nodules diagnosis.

Finally, we evaluated the clinical applicability of thyroid nodules diagnosed using AI-assisted ultrasound diagnosis technology. The results showed that when the pre-test probability was set at 30.00%, the positive likelihood ratio (PLR) was 6.00, and the post-test probability of a positive test result reached 71.00%. The NLR was 0.13, and the post-test probability for negative test results was only 5.00%, indicating that the thyroid AI-assisted diagnostic system has high clinical predictive value (**Figure 8**). Diagnostic performance was visualized using a likelihood ratio scattergram, where PLR < 10.00 and NLR > 0.10 indicate higher diagnostic accuracy. The combined effect size of all diagnostic models showed that the intelligent auxiliary diagnostic model performed poorly in terms of diagnosing and ruling out malignant thyroid nodules. However, as shown in the figure, the intelligent diagnostic models used in the five studies (11, 13, 14, 18, and 23) demonstrated very high accuracy for thyroid nodules, with the models used being PLS-DA, AIBx V2 (DCNN, ResNet 34), DCAD (FCN, DCNN), EDLC-TN (DCNN, DenseNet), and XGBoost (**Figure 9**). After comprehensively comparing the sensitivity, specificity, DOR, PLR, and NLR of these five models, we found that the model used in the WeiX2020 (23) study had a sensitivity of 0.93 (95% CI: 0.90–0.96), specificity of 0.94 (95% CI: 0.92–0.96), Diagnostic Score was 5.37 (95% CI: 2.67–5.37), Odds Ratio was 215.25 (95% CI: 127.73–362.73), DLR Positive was 15.44 (95% CI: 11.33–21.03), and DLR Negative was 0.07 (95% CI: 0.05–0.10) (**Figures 4A–C**). The model used was EDLC-TN (an ensemble deep learning classification model for thyroid nodules). The ROC curve for the AI-based diagnosis of thyroid nodules showed an AUC of 0.93 (95% CI: 0.91–0.95), indicating that the thyroid intelligent assistance model has high accuracy in distinguishing between benign and malignant nodules (**Figure 3**).

## 4 Discussion

In recent years, artificial intelligence tools have become increasingly common in various disciplines. Artificial intelligence is beneficial in assessing thyroid nodules, primarily for risk stratification and the diagnosis of benign and malignant thyroid nodules. This meta-analysis encompassed a substantial number of studies that examined various types of AI models, spanning from the past to the present. Our goal is to systematically compare the performance of existing artificial intelligence models in identifying benign and malignant thyroid nodules based on ultrasound image features, identify the optimal model, and provide guidance for developing improved intelligent diagnostic models. We systematically investigated sources of heterogeneity and compared the I2 for subgroup analyses with the pooled effect size, I2, which showed no significant changes in sensitivity or specificity across subgroups. Given the obvious heterogeneity suggested by I2 after the combined effect size, we also performed univariate and multivariate meta-regression analyses for different subgroups. All factors except region were sources of heterogeneity, among which the malignant rate of thyroid nodules, and different AI types were significant sources of heterogeneity. At the same time, the diagnostic accuracy of AI models is constrained by multiple factors, all of which increase the complexity of diagnostic model construction (34, 35).

### 4.1 The quality of ultrasound images

Ultrasound imaging provides standardized, high-contrast visual features of thyroid nodules, including size, shape, density, and blood flow signal patterns, on which both artificial intelligence algorithms and radiologists rely for assessment. The lack of a uniform ultrasound image acquisition protocol across the original studies included in this study, coupled with the variability in institutional operating procedures and equipment specifications (E.g., Ransducer frequencies, gain settings), greatly limits the generalizability of the current model across different clinical Settings. The frequency of the ultrasound probe will affect the resolution of the image; the higher the frequency, the higher the resolution. Different ultrasound devices and probes were used in each original study, which is also a source of potential heterogeneity. For example, in the study of ChenJH et al., L14-3U transducer of Resona 9 device (Mindray, Shenzhen, China) (frequency: 3–9 MHz) and an L12–5 transducer (frequency: 5–12 MHz) from an iU22 device (Philips, Amsterdam, The Netherlands) (9). However, KimYJ et al. used a higher frequency probe, and the Philips iE33 US instrument with a 12–15 MHz linear array transducer (Philips Medical Systems) provided ultra-high resolution imaging for image acquisition (12). A multicenter study showed that ultrasound equipment from different manufacturers had a significant impact on AI model performance. Studies conducted in Asia may have overrepresented certain regional manufacturers (e.g., Mindray) and underrepresented brands commonly used in the West (e.g., General Electric) (36). In addition, the number of phantom ultrasound devices included in each study varies greatly, which can significantly affect the image quality. YaoJC et al. collected ultrasound images of patients using 26 different devices produced by the General Electric Company, Philips, Esaote, Siemens, and Toshiba (25). Heterogeneity of ultrasound systems and operators from different manufacturers may contribute to variability during training. Of course, different types of ultrasound devices were used in the study, which helped to increase the diversity of the data.

Almost all studies were trained and tested on only a representative single image of each nodule, and the reviewed images were static two-dimensional images, ignoring dynamic features such as hemodynamics and tissue elasticity. Most studies have selected transverse and longitudinal sections, while other sections are rarely included. The selection of representative images and semi-automatic segmentation can theoretically be affected by the operator’s experience, and this method may limit the diagnostic ability of observers and artificial intelligence models. For example, Buda et al. used two orthogonal images of transverse and longitudinal sections as model feature extraction images (6). In clinical practice, physicians often rely on real-time ultrasound information to evaluate thyroid nodules rather than a single representative image. Thus, radiologists were able to assess based on more thorough imaging of each thyroid nodule, which may explain the higher performance of experienced radiologists. At present, due to the complexity of 3D model architecture, the limitation of computational efficiency, and the difference in tools, 3D dynamic image models have not become a research hotspot. At present, researchers have developed artificial intelligence models based on contrast-enhanced ultrasound (CEUS) images. For example, the study by ChenJH et al., included in this study, applied machine learning to extract radiomics features of two-dimensional ultrasound (2D-US) combined with contrast-enhanced ultrasound (CEUS) images of the thyroid to classify and predict benign and malignant thyroid nodules, with an AUC of 0.94 (9). In addition, manually labeled ultrasound images can significantly affect the accuracy of the model. The images mainly extract image Texture Features, Higher-Order Features, and other information, and the labeled images affect the model’s ability to learn features, especially small nodules. For example, the study of ZhuJL et al. included marked thyroid nodule images (33), which is also a potential source of heterogeneity and an important factor affecting the accuracy of the model.

### 4.2 Size of thyroid nodule

Different nodule sizes can affect the recognition ability of AI diagnostic models. In this study, the diameter sizes of nodules in different original studies were quite different. In general, for image feature extraction, the degree of thyroid nodule margin of large nodules cannot be judged because it exceeds the view boundary, which may lead to poor model performance. Chen et al. showed that nodule size and tumor edge roughness were risk factors for detecting malignant nodules (8, 16, 37, 38). This information will be lost when the nodule is too large. However, for small nodules, especially thyroid micropapillary carcinoma smaller than 10mm, the boundary may not be clear, and the AI system may not have enough identifiable features to make a correct prediction. The study by KoSY et al. showed that the inclusion of thyroid nodules of different sizes would affect the results, and their model findings could not guarantee high accuracy in other studies with large groups of different nodules (14). An international study shows that in Asia, more thyroid cancers are detected through asymptomatic screening (51%), whereas in Western countries, more cancers are detected through symptoms (30%). Nodules detected by screening are usually small and have atypical features, which may lead to inconsistent model criteria for small nodules in different regions (39). In addition, the biopsy rate of small nodules is low in clinical practice. According to ATA guidelines, most thyroid nodules less than 10mm are treated conservatively, which leads to insufficient data in the training set and is also a factor causing bias (37).

### 4.3 Different subtypes of thyroid nodules

Diagnosis of thyroid nodules is not only about identifying benign and malignant nodules, but also requires accurate identification of benign and malignant nodules of various histologic types of the thyroid gland. Existing training datasets are often inadequate in size, especially for rare histologic subtypes such as follicular carcinoma, which may introduce selection bias and affect model performance. This meta-analysis study included only two (25, 28) that sampled follicular thyroid carcinoma, and there are still fewer studies on rare subtypes, and determining the specific type of follicular thyroid nodule is a difficult challenge in accurately diagnosing thyroid cancer. Fine needle aspiration cytology (FNAC) can only provide a vague Bethesda class IV diagnosis with uncertain malignancy and clinical prognosis, most models only included nodules with malignant or benign clear pathologic findings, and most studies excluded thyroid nodules that were undiagnosable or cytologically or histologically indeterminate, such as Bethesda class IV suspected malignant nodules.YaoJC et al. constructed the swin-transformer (ST) model for follicular thyroid nodules with an AUC of 0.90 (25). In the dataset studied by ParkVY et al., 95.5% of the patients had typical papillary thyroid carcinoma with ultrasound features different from those of follicular carcinoma, medullary carcinoma, and lymphoma. However, other subtypes, such as follicular carcinoma and mesenchymal carcinoma, also had some different ultrasound features compared to benign thyroid lesions. The algorithm may be less efficient in diagnosing medullary carcinoma compared to other subtypes because it has more ultrasound features compared to benign thyroid lesions. Therefore, there is still a need to validate the diagnostic model for nodules with indeterminate cytological findings and therefore characterized as “heterogeneous of uncertain significance” or “follicular lesions of uncertain significance” (19). The proportion of papillary thyroid carcinoma may be higher in Asian populations than in the West, whereas other subtypes, such as follicular carcinoma, are relatively rare (40). Since the majority of malignant nodules in the current studies were papillary carcinomas, it is still not possible to evaluate and compare the performance of machine learning algorithms in each cancer subtype. In addition, Hashimoto nodules and chronic atrophic nodules can exhibit similar ultrasound features to malignant nodules, and there are differences in the ultrasound features of different pathologic types, which may decrease the classification performance when the model is applied to regions with different pathologic spectrums (41).

### 4.4 Regional differences

About 89% of the subjects in this Meta-analysis were from Asia, and only 11% were from Western countries. Although most studies verified the results in different external test sets, the accuracy and clinical applicability of the results were satisfactory; however, there was inevitably racial bias, and this regional difference may be one of the important sources of heterogeneity in the studies. In a statistical study of 25 countries, the incidence of thyroid cancer in Asian countries was significantly higher than that in Western countries, mainly because the popularization of ultrasound screening in Asian countries led to overdiagnosis, especially in middle-aged and elderly individuals older than 50 years, rather than an increase in true disease (39). The increase in incidence may also be related to economic development, population life pressure, inflammation, and other factors. Asian countries have invested heavily in thyroid disease research and accumulated a large number of high-quality thyroid ultrasound image datasets. After rigorous screening and annotation, these datasets provide a solid foundation for model training, enabling the model to identify the characteristics of thyroid nodules in this population more accurately. A recent Chinese study was trained and tested on ultrasound data from nine hospitals (42). Researchers in Asian countries have optimized and adjusted the diagnostic model according to local medical practice and patient characteristics. By adjusting the model’s parameter settings and refining the feature extraction method, the model becomes more suitable for the medical environment and patient population of Asian countries, thereby improving diagnostic accuracy. It may not perform well in Western populations, particularly in thyroid cancer subtypes with distinct distributions. This is consistent with the results of the subgroup analysis of this study, showing that the diagnostic accuracy of the AI diagnostic model was higher for Asian countries. However, the results of subgroup analysis in this meta-analysis showed that the effect of country and region subgroups on heterogeneity was not statistically significant, possibly due to the small number of studies included in Western countries.

### 4.5 Feature extraction methods

According to the 2015 American Thyroid Association ATA guidelines, it is divided into six types, including mixed type, intermediate type, and peripheral type, only in terms of calcification. At present, suspicious ultrasound features of thyroid cancer are mainly identified by sonographers, and accurate differentiation of these features is difficult and may be misunderstood or missed (37). Feature extraction is primarily divided into two approaches: experienced radiologists and radiomics. The former is based on human feature extraction, while the latter is based on computer-based image analysis. AI models are typically trained on datasets annotated by experienced radiologists, essentially learning to recognize the same diagnostic patterns as those used by human experts (43). In traditional machine learning models, experts in the relevant domain will first set the most applicable features to reduce the complexity of the data and highlight patterns. However, some hidden relationships may be lost when manually entered, and they may not be able to capture complex, non-intuitive relationships in the data in advance. However, these features may be hidden in a simple image texture, and computer-aided diagnosis (CAD) systems are a novel AI technique developed to achieve automatic analysis of ultrasound images (44). ZhaoCK2021, ZhengYX2024, and other studies included in this study all manually extracted ultrasound features (27, 28), while ChenJH2024, LaiM2023, and other studies automatically extracted features through CNN (9, 15). By comparing the feature extraction methods, we found that ultrasound image features extracted by radiologists were significantly better than image-based methods, such as those used by Chen et al., who extracted empirical features by radiologists for TI-RADS classification, with an AUC of 0.97 (8). However, the model of Lai et al. used the PyRadiomics (45) feature extraction package to extract radiomics features for training machine learning models, and its AUC was only 0.856, which was significantly lower than the empirical features extracted from ultrasound images (15). Features automatically extracted by computer-based image analysis models may contain redundant information (such as artifacts in ultrasound images). Instead of selecting radiomics features with partial thyroid nodule signs in a single image, an experienced radiologist reviews a series of thyroid ultrasound images to make the final decision. It has the best correlation with the nature of the nodule. Traditional methods (such as logistic regression + manual features) can explicitly incorporate clinical prior knowledge (such as Bethesda classification criteria), while AI model extraction of features may ignore these key rules (46). Furthermore, the large number of different radiomics features poses an intractable challenge that hinders the use of machine learning models for diagnosing thyroid nodules in the clinic (47).

A recent study by Chen JH et al. used a machine learning model (DT) to construct feature importance combined with LASSO to select features (9). With different feature screening methods, the final remaining features are different, and the accuracy of the final model is also different. Although the malignant characteristics of different types of TI-RADS, such as C-TIRADS and ATA-TIRADS, are basically the same, there are also differences. There may be differences in the interpretation criteria of ultrasound features by radiologists in different regions. When a standard training model for a single region is used, performance may decline in other regions (48). The different sections of thyroid nodules, such as longitudinal section and transverse section, will affect the extraction of ultrasound image features. The number of representative ultrasound features used in each study varied; for example, the study by Chen et al. (8) included 10 ultrasound features, while the study by zhaoCK et al. (27) included only 6 ultrasound features. Finally, the image acquisition process of KoSY et al. ‘s study, such as selecting input images for thyroid nodules and labeling ROIs, relied on radiologists and was essentially operator dependent due to the perception variation of radiologists and the possibility of ignoring obvious features (14). Aspects of the influencing characteristics are a source of potential heterogeneity and also limit further exploration of potential effects on diagnostic efficacy.

### 4.6 Malignant rate of thyroid nodules

The results of this study showed that the higher the malignant rate of thyroid nodules, the stronger the diagnostic performance of the AI diagnostic model. However, most of the included studies used retrospective data, which was susceptible to case selection and information bias. Additionally, the cases in both the training set and test set were all patients who underwent thyroid ultrasound screening in hospitals. As can be seen from the extracted data table, the proportion of malignant thyroid nodules in each of the included original studies was significantly higher than that in the normal population (8, 30). The study by ChenYF et al., which included thyroid nodules with surgical resection or FNA, had a high overall malignancy rate, and only thyroid nodules with surgical pathological findings were included, which may have resulted in a higher than usual rate of malignant nodules (10). The real-world data would be a greater proportion of benign nodules and a smaller proportion of malignant nodules, which could lead to overfitting and thus compromise accuracy. The surgical rate of small thyroid nodules (<1cm) in Asian countries (such as China and South Korea) is significantly higher than that in Western countries, and there is a higher proportion of overdiagnosis. This difference leads to a different proportion of “surgically confirmed benign nodules” in the training data, and the proportion of malignant cases in the training data is distorted, which affects the model’s judgment threshold for the true malignant risk (40). Additionally, thyroid nodules have a high risk of malignancy, which may have contributed to selection bias in our sample. Since the cytologic results of fine-needle aspiration biopsy may be inconclusive, only the histopathological results of surgical resection can be used as the reference standard for the final diagnosis of nodules.

In our meta-analysis study, 11 studies were identified with case selection bias, as the original authors had selected only surgical patients. According to the 2015 American ATA guidelines, patients with thyroid nodules, if they are highly suspected of malignancy or have been confirmed to be malignant and the Bethesda reporting system indicates category V or VI, even in low-risk Papillary Thyroid Microcarcinoma(PTMC) patients, surgical treatment is not recommended (37). However, these 11 studies included patients who did not undergo FNA after ultrasound evaluation and chose to proceed directly to surgery. The surgical criteria were outlined as follows: benign nodules exceeding 4.0 cm in size and malignant nodules confirmed by preoperative needle biopsy pathology. For cases where ultrasound strongly suggested malignancy but the standards for fine-needle aspiration biopsy (FNAB) weren’t met, FNAB was advised prior to determining subsequent management (22). Therefore, we considered that the patients were highly suspected of malignancy, such as a history of thyroid radiation in childhood, family history of thyroid cancer, extrathyroidal extension, and thyroid ultrasound assessment of TI-RADS classification 4c or 5. We considered that there was a high risk of patient selection bias in these studies, so only surgical patients were selected as the sample. It was rated as high risk in terms of patient selection. Because the original authors evaluated the models using a validation set, and both achieved high diagnostic accuracy, they were still rated as low risk in terms of Applicability Concerns.

### 4.7 Age of patients

Additionally, this study found that the AI-assisted diagnosis system demonstrated higher diagnostic performance for patients aged 50 years or older. Results such as those of Chen et al. suggest that age is an important risk factor associated with malignancy. The prevalence of thyroid nodules gradually increases with age, which is consistent with previous research results (8, 49). Age-related tissue degeneration, fibrosis, or calcification may change the ultrasonographic appearance of nodules. Older patients often have multiple coexisting lesions that may interfere with image interpretation, and age-related histological changes such as fibrosis and calcification may affect the ultrasonographic features. As a result, the model’s performance fluctuates when applied across regions, affecting its accuracy (50).

### 4.8 Gender of patients

The detection rate of thyroid nodules in females is higher than that in males, which is a risk factor for the detection of thyroid nodules. The increasing incidence of thyroid cancer among women has been particularly marked since 2000 (39). The results of this study showed that the larger the proportion of women ≥70%, the higher the intelligent assisted diagnosis system’s sensitivity was, at 0.90 (0.87-0.92), and specificity, at 0.85 (0.81-0.89), in the diagnosis of thyroid nodules, (50). The effects of estrogen may be the primary factor contributing to the incidence of thyroid nodules in women. In this study, the majority of the included original studies showed a significantly higher proportion of female patients than male patients, which made the model training more biased toward females. However, in external validation sets such as those in Chenc et al.'s study, the proportion of male patients was significantly higher than that of female patients, which could also affect accuracy (7, 51). The proportion of female patients was positively correlated with AI accuracy, but due to the limited number of original studies with a higher proportion of male patients, gender-specific conclusions still require further validation.

### 4.9 Reference standards

The gold standard is the reference standard for the accuracy evaluation of diagnostic tests. It must be the most recognized and accurate diagnostic method at present, and its implementation, testing timing, methods, and interpretation standards should be unified. If the gold standard is interpreted by different people, it may lead to differences in the results and thus introduce bias. The different exclusion and inclusion criteria of each of the included original studies were also a potential source of heterogeneity; the study by ChenJH et al. excluded patients with incomplete FNA pathological results or classified as Bethesda I, III, or IV. However, Koh et al. included only surgically confirmed or cytologically confirmed benign (class II) or malignant (class VI) on the Bethesda system. Different reference standards affect the diagnostic accuracy of AI models (9, 13).

### 4.10 Model architecture

Notably, compared with traditional machine learning methods, deep learning demonstrated higher accuracy in diagnosing thyroid nodules, with a sensitivity of 0.89 (0.87-0.91) and a specificity of 0.85 (0.80-0.89). Traditional machine learning models often use a variety of classifiers in the development process, such as the study of JinZ et al. (11) using random forest (RF), support vector machine (SVM) and extreme gradient boosting (XGBoost) to build ultrasound and ultrasound and clinical combined machine learning models, respectively. Different classifiers have their characteristics. The decision tree (DT)-based methods achieve classification and regression tasks through conditional probability distributions, but DT, as a non-parametric method, is prone to overfitting. However, DT-based ensemble methods (such as random forest, RF; gradient boosting decision tree, GBDT; and XGBoost) significantly improve performance. By randomly selecting feature subsets and samples for parallel training, RF has the advantages of high training efficiency, low generalization error, strong noise resistance, and simple parameter adjustment. GBDT enhanced feature selection ability by gradient boosting (52). XGBoost provides further support for custom loss functions, regularization terms, and handling of missing values, demonstrating greater flexibility. In contrast, linear models have obvious limitations: Support vector machine (SVM) can deal with nonlinear problems through kernel functions, but the selection of kernel functions is time-consuming and difficult to adapt to large-scale data. Although logistic regression (LR) is suitable for multi-classification tasks with small samples, its function is limited. Overall, RF and XGBoost significantly exceed the performance boundaries of traditional DT and linear models with their stability and flexibility, respectively. These traditional models are built on the paradigm of “artificial features + shallow models”, and their performance highly depends on the quality of feature engineering. Features automatically extracted by deep learning (DL) models may contain redundant information (such as artifacts in ultrasound images). Whereas traditional methods (such as logistic regression + manual features) can explicitly incorporate clinical prior knowledge, DL models may ignore these key rules (46). Although the SVM model can achieve nonlinear classification by kernel function, feature extraction completely relies on manual design, which faces problems such as a high threshold of expertise, limited dimension, and feature interaction. The shallow nature of the model structure also limits the expression ability. Linear models can only learn global linear relationships. Although DT can capture nonlinear features, the tree structure based on the greedy algorithm is easy to overfit and ignore the global pattern. These fundamental defects make traditional machine learning perform poorly in processing high-dimensional and complex data such as images and speech, highlighting its inherent limitations in feature learning and deep pattern capture.

Deep learning algorithms consist of structures called deep neural networks, of which convolutional neural networks are a specific type that is widely used in the field of image processing (53). Its multi-threaded task can be used for more complex radiomics image analysis. For example, Zhou et al. ‘s study adopted a CNN architecture and a transfer learning strategy and achieved a high AUC of 0.97 (29). The study by Yao et al., which constructed a multimodal deep learning model involving 6,032 (Papillary Thyroid Cancer, PTC) cases, was used to develop the DeepThy-Net model, which showed high accuracy (54). Lee, J.H., et al. used deep learning to locate and diagnose metastatic lymph nodes in thyroid cancer and achieved high clinical application value (55). These findings collectively demonstrate the excellent diagnostic performance of artificial intelligence, regardless of imaging type, and the emergence of deep learning as a more powerful approach to diagnosing thyroid cancer. Deep learning has an inherent architectural advantage. The deep learning system trained by pre-simulation can automatically obtain relevant ultrasound features through multi-level nonlinear transformation. The hierarchical feature learning capability of deep learning enables the automatic extraction of complex multi-layered imaging features from raw ultrasound data, whereas machine learning relies on predefined features that may miss subtle details (53). For example, a convolutional neural network (CNN) has a layered architecture that mimics a biological vision system: the bottom layer learns local features such as edges and colors, the middle layer combines textures and parts, and the upper layer forms a complete object representation. This end-to-end hierarchical feature learning has the advantages of distributed encoding and incremental refinement, enabling the model to automatically extract more essential feature representations from the data. Deep networks such as the Transformer architecture can model long-distance dependencies in natural language through a multi-layer self-attention mechanism, and achieve the same expressive ability with fewer parameters. The superior performance of deep learning also stems from its ability to learn complex spatial relationships and identify relevant features, especially in analyzing the complex anatomy of thyroid nodules and surrounding structures (56). The data-dependent nature of deep learning algorithms shows that model performance continues to improve with the increase in the amount of training data, while classical machine learning algorithms tend to be stable. Meanwhile, most well-performing deep learning models are generated from baseline architectures, and the diagnostic performance of AI models will be further improved through innovative modifications of training strategies and algorithm architectures (19, 29). As more and more comprehensive features are included in artificial intelligence models, their prediction results will be closer to pathological results. Deep learning models, such as DCNN, can learn features directly from ultrasound images without the limitations of manually designed features, thereby improving the repeatability of diagnosis.

### 4.11 External validation set

External validation was designed to explore true differences in characteristics between the development and validation cohorts and to evaluate the performance of diagnostic models. All selected studies were conducted strictly according to the independent external validation set, except for VelascoPF2024, which used the same data set for both internal and external validation. However, some of the studies included in the literature utilize public data sets (such as the Stanford dataset) as internal data sets, which is questionable (18, 21). Although this is a practical option, the overall characteristics of these open-source data sets are often difficult to obtain and limited by inadequately collected data. Diagnostic models generally perform well on training datasets; however, there are differences between the performance of the model on the training set and the performance on the validation set. This difference may be reduced when the training and validation datasets are drawn from the same population, and this imperfect model training and validation result in poor model performance on the external validation set. The results of this study showed that the diagnostic performance of the AI model in the non-cross-validation group was higher than that in the cross-validation group, mainly because the sample size of each study in the non-cross-validation group was much larger than that in the cross-validation group, and more patients’ nodules were included in the model, which eventually had better test power and generalization. Due to the lack of large-scale external validation, most AI applications have not yet been applied to clinical decision-making. Therefore, external validation is necessary to reduce the risk of model overfitting, assess the stability of model performance in different populations, and potentially enhance clinicians’ confidence in AI-assisted diagnostic tools.

### 4.12 Prospective study

In prospective studies, the performance of AI-assisted diagnostic techniques, particularly in terms of sensitivity, specificity, and the area under the curve (AUC), is outstanding. Prospective studies can be enrolled at the onset of symptoms and performed according to prespecified diagnostic criteria and trial algorithms, reducing selection bias. However, retrospective studies may have problems, such as selectivity, including the retrospective selection of patients who have been diagnosed, which can miss some undetected cases and lead to bias. However, only two prospective studies were included in this meta-analysis, accounting for only 7% of the total studies, which may affect the accuracy of the results (19, 30).

In addition, this meta-analysis included various artificial intelligence models, including some commercial computer-aided diagnosis (CAD) and machine learning (ML) models. Although several commercial AI-assisted diagnostic systems have been introduced into clinical practice in recent years, such as the AI-SONIC™ Thyroid model mentioned in the ZhouTH2024 study, which achieved a sensitivity of 95% for identifying malignant thyroid nodules, some manufacturers may adjust the model thresholds to improve sensitivity, which could affect the accuracy of the results in this study. The heterogeneity of imaging systems and operators across different manufacturers may introduce variability during the training process, potentially limiting the interoperability of devices manufactured by other suppliers (30, 57). However, due to differences in algorithm architecture, training dataset quality, and validation methods, significant disparities remain in the diagnostic performance of these systems, and their clinical application value requires further validation and optimization (30).

The bivariate boxplot results of this study suggest significant heterogeneity among the four articles, specifically studies 1, 15, 16, and 28 (**Figure 5D**). Although this does not affect the reliability of the final pooled effect size, we carefully analyzed these four articles from various aspects, including population characteristics, diagnostic thresholds, reference standards, study design, and sample size, to identify the specific reasons for the outlier studies. We found that studies 1 and 16, BudaM2019 and VelascoPF2024, used cytopathology from fine-needle aspiration (FNA) as the sole reference standard and had lower malignant rates of included nodules compared to other studies, at 10.3% and 11.1%, respectively. Consequently, the specificity for diagnosing thyroid nodules was only 52.0% (6, 21). We analyzed studies 15 and 28, SunC et al.’s DCNN (VGG-F) model, which actually combines multiple features, using SVM for feature extraction, and combines the deep features prioritized by the convolutional neural network (CNN) with hand-crafted features (20). ZhuYC2022 et al.’s ANN (TDUS-Net) model employs color Doppler ultrasound (CDUS) features extracted via deep learning (whole ratio, intranodular ratio, peripheral ratio, and number of vessels) and gray-scale ultrasound (US) features, which differ significantly from those of other models (33). These factors may be a significant source of heterogeneity.

Ultimately, we selected the WeiX2020 study as having the optimal model. The core advantage of the EDLC-TN model lies in its innovative ensemble learning framework and multi-stage design (23). The model first extracts the region of interest (ROI) through precise nodule segmentation, eliminating interference from irrelevant background in ultrasound images, so that subsequent classification can focus on key nodule features (such as edge morphology and internal echoes). In the classification stage, EDLC-TN employs a multi-model ensemble strategy based on DenseNet (58): by training three structurally distinct weak classifiers (based on ROI, mask, and fused features, respectively) and integrating their outputs via voting and averaging methods, the model retains DenseNet’s efficient capture of subtle features through dense connections while addressing the limitations of single algorithms through model diversity. This design enables the model to achieve an accuracy rate of 98.51% (AUC 0.941) on the test set, far surpassing other comparison models. Additionally, the superiority of EDLC-TN is evident in its data and training strategies. The study utilized a multi-center dataset (26,541 images spanning four hospitals and various devices), implemented dynamic learning rate adjustment (Adam optimizer gradually decaying from 0.1), and enforced strict data standardization. In external validation (unseen GE ultrasound device-acquired image data), the model maintained an accuracy rate of 95.76%, demonstrating its cross-device adaptability. The constructed EDLC-TN model is a universal network platform that can be applied to ultrasound images from different medical centers. Whether applied to ultrasound images from hospitals with completely different types of ultrasound devices or compared with the performance of radiologists, the model achieved excellent accuracy, sensitivity, and specificity. This indicates that the EDLC-TN model has the potential to effectively learn from different types of medical images and possesses high generalizability. This will be beneficial for screening programs and lead to more effective referral systems across all medical fields, thereby yielding widespread beneficial clinical and public health outcomes. However, due to the limited number of studies on which the model is based, its generalizability and applicability across different medical centers require further validation through large-scale, multi-center prospective studies.

Additionally, interpretability models remain a significant research challenge. At present, many scholars have doubts about the reliability of AI models. As a “black box” system, these models lack the interpretability of the diagnosis and decision-making process, resulting in a cognitive gap between doctors and models and weakening clinical trust. Interpretable AI is a set of tools and methods that help people understand and interpret the predictions made by machine learning algorithms. This includes an interpretable model and an interpretable interface. This contributes to the accuracy, fairness, and transparency of diagnostic models and understanding of the results of AI-driven decision making (59). The reliability and interpretability of diagnostic AI tools can be improved through collaborative engagement between AI developers and clinical practitioners. In addition, most models only output static binary classification probability results (such as malignant probability) and lack an interactive feedback mechanism with clinicians. This “silent box” feature makes doctors more inclined to use traditional transparent diagnostic methods, which leads to doubts about the actual value of AI and hinders the deep application of big data technology in the medical field (8, 26). In the study of Zheng et al., the model was constructed, and the interpretability analysis was performed through the Shapley Additive Explanations (SHAP) to achieve the transparent effect of the model (28). The study by Yao et al. developed a multimodal generative pre-trained transducer model, ThyGPT, which provides a transparent, interpretable, publicly available, and efficient AI-aided tool for the diagnosis and management of thyroid nodules (42). Model transparency requires further optimization to explain the decision-making process of algorithms, and the accurate conclusions of deep learning models still need to be verified on external test sets with large samples (60–62).

The artificial intelligence system offers a new option for diagnosing thyroid nodules and has significant clinical application value. This meta-analysis presents a comprehensive subgroup analysis of AI models, addressing emerging trends in imaging technology. These methodological advances contribute to a more complete understanding of the role of ultrasonographic AI in the diagnosis of thyroid nodules, although further validation in different populations is still needed. AI diagnostic models (especially deep learning algorithms) can significantly improve the diagnostic accuracy and sensitivity of radiologists at all levels (63), which can effectively improve the diagnostic accuracy of young radiologists and radiologists in primary hospitals, and shorten the growth cycle of radiologists. Urban-rural medical resources are not balanced in China and many countries worldwide. The research and development of artificial intelligence systems can help reduce barriers and provide convenient ways for community hospitals to improve the diagnosis of thyroid cancer. The implementation of ultrasound-based AI in the primary healthcare system could enable the early detection and management of thyroid nodules, particularly thyroid cancer, in remote areas with limited diagnostic resources. AI models can also reduce the time required for image interpretation by doctors, decrease clinical workload, decrease labor intensity, and mitigate the influence of subjective factors on diagnosis. Additionally, they can serve as a reference for clinical experts to enhance the quality of clinical diagnosis of thyroid cancer, particularly for smaller thyroid nodules (64). AI technology can efficiently extract image features, transform the traditional image diagnosis from subjective qualitative analysis to objective quantitative assessment, and realize the whole process of auxiliary diagnosis from nodule detection, benign and malignant judgment to pathological classification and prognosis prediction, which is helpful to promote the transformation from population to individual diagnosis and treatment of thyroid nodules (65). If widely and continuously implemented, it is expected to reduce the relatively invasive FNA biopsy in the diagnosis of thyroid nodules, avoid the discomfort of patients in the process of FNA biopsy and the risk of biopsy-related side effects or complications, realize the early and accurate diagnosis of thyroid nodules, improve the influence on treatment options and prognosis, reduce the risk of delayed diagnosis caused by false negative results, and reduce the anxiety of patients. Reduce healthcare spending and improve patient satisfaction and patient experience. The use of AI models did not lead to a change in the mortality rate despite an increase in the detection rate of thyroid cancer. The medical community also needs to pay attention to the problem of overdiagnosis. For example, it may be more appropriate to use AI models to adopt active surveillance strategies for small papillary carcinomas. In the future, the workflow optimization of AI initial screening and physician confirmation, as well as dynamic monitoring integrated into portable devices, can help doctors dynamically monitor disease progression and optimize the management of high-risk patients, which is expected to further improve the accuracy and accessibility of thyroid nodule management.

In the actual clinical scenario, when physicians need to perform a comprehensive evaluation of patients with thyroid nodules, they may encounter a large amount of information from case data, clinical features, radiomics, and genomics. It is important to study the ability to build a unified model from both macro and micro levels, integrate and analyze information such as pathology, genomics, and clinical data, so as to achieve truly multimodal and cross-body assisted diagnosis. At present, diagnostic models based on thyroid ultrasound image features are gradually favoring hybrid models and multimodal large language models. For example, the weights of the hybrid assisted multi-classification model consisting of Deep Maxout and CNN built by Gulame et al. are automatically learned through transfer learning, which is significantly better than the earlier method of a single model (66). Yao et al. proposed an AI-assisted diagnosis system of a large language model for thyroid nodule risk assessment. The ThyGPT model was trained and verified on large sample data sets, and the LlaMA2-13B model framework was used to segment and store thyroid-related knowledge and cases combined with the Lang-Chain framework. The hybrid architecture of Swin-Transformer and DCNN is used for image analysis, which integrates multi-source information to generate feature markers, and intuitively displays the decision-making basis and the contribution of ultrasound features through human-computer interaction. This model provides a new direction for the development of next-generation CAD systems (67).

Despite the advantages of AI models, enormous challenges remain. First, the use of artificial intelligence models in clinical practice raises ethical and legal issues. Regulators must develop clear guidelines that address issues such as patient privacy, data security, and diagnostic decision-making responsibility to ensure the safe, effective, responsible, and ethical use of AI technologies in clinical Settings. Addressing these challenges is critical for the successful implementation and widespread adoption of auxiliary diagnostic models for thyroid nodules in clinical practice. Second, future AI developments should prioritize adaptability, algorithmic rhythm transparency, and interpretability to facilitate professional acceptance and integration of AI-assisted diagnostic technologies. At the same time, unresolved issues regarding data security protocols and ambiguous accountability frameworks in smart healthcare technologies continue to impede clinical adoption. Third, at present, AI is more suitable for use as an adjunct rather than a stand-alone diagnostic method, and its results must be reviewed by certified physicians. Further research and method standardization are needed to address these discrepancies in the future, to integrate AI software seamlessly into physicians’ workflows, and external validation studies with large samples should be conducted (68). Fourth, clinical ultrasound is a highly dynamic diagnosis and treatment process, and doctors rely on real-time images for comprehensive judgment. Dynamic data processing will face many technical challenges. The amount of data to be processed by real-time video analysis is hundreds of times that of static images, which requires the development of new neural network architectures (such as 3D CNN+Transformer). Instead of the current mainstream 2D CNN model, the inference delay needs to be controlled to ensure clinical usability, which puts forward extremely high requirements for algorithm optimization. A lightweight model (such as deformable convolution) specifically for the characteristics of ultrasound video is developed, which integrates color Doppler, pulse wave, and other multimodal timing data, and the chip inside the ultrasound probe realizes end-to-end real-time processing. In the future, dynamic ultrasound database standards (such as probe motion trajectory and time synchronization markers) will be established. Evaluation frameworks such as real-time human-computer interaction tests will be developed through databases to simulate clinical environments. Progressive verification processes will be designed from static frames to video clips, and then stepwise verification of complete clinical scenarios will be formed through real-time streaming. Finally, international multicenter data sets should be established in the future to ensure that the samples of each subgroup are balanced, include data from different ultrasound devices and different acquisition protocols, and adopt unified image annotation standards and pathological confirmation procedures to construct diagnostic models for different subtypes of thyroid nodules. The model needs to create a well-defined set of features that can be efficiently captured by human visual assessment and computational analysis (69). The model can incorporate the characteristics of cervical lymph nodes and also provide value for surgical methods used in treating thyroid cancer patients, such as lymph node dissection. AI models offer promising prospects for clinical applications, and factors such as human selection bias and reliance on human labor can be improved.

This meta-analysis should acknowledge several important limitations. First, the heterogeneity among the included studies is significant. In addition to the various factors discussed in the analysis section, there may be some unrecognized factors that have been overlooked or that could not be further analyzed due to the limited number of original studies. Second, over half of the studies employed retrospective designs, which may introduce potential selection and bias. Since researchers developed AI models, the training phase required the construction of models with accurate labels based on reference standards, a step that necessitated a retrospective design, whereas the predictive phase, which involved a validation set (or test set), did not. Third, the generalizability of these findings may be limited by the fact that most studies were conducted in Asian regions, so our findings may have limited generalizability to populations in other regions. Fourth, since some basic characteristics of thyroid nodules, such as nodule echogenicity, internal structure, and calcification, were not included, it is not possible to further explore the impact of these basic characteristics on diagnostic performance. Finally, we only included studies written in English, which may make this meta-analysis more susceptible to publication bias.

## 5 Conclusion

This meta-analysis examined the clinical value of an artificial intelligence-assisted diagnosis system based on ultrasound images in the diagnosis of benign and malignant thyroid nodules, and the results demonstrated high clinical potential. Among them, the EDLC-TN model shows higher diagnostic accuracy and clinical effectiveness in the diagnosis of thyroid nodules. For thyroid nodules in female patients with an average diameter of <20mm and an age of ≥50 years, artificial intelligence-assisted diagnostic models are more effective, especially deep learning models.

## Data Availability

The original contributions presented in the study are included in the article/**Supplementary Material**. Further inquiries can be directed to the corresponding author.
