# Supplementary material for: Diagnostic performance of ultrasound characteristics-based artificial intelligence models for thyroid nodules: a systematic review and meta-analysis
Source: Front Oncol. 2025 Sep 3;15:1614603. doi: 10.3389/fonc.2025.1614603 (PMC12440764; doi:10.3389/fonc.2025.1614603)
Supplement: Supplementary file 1 [file DataSheet1.docx]

Diagnostic Performance of Ultrasound Characteristics-Based Artificial Intelligence Models for Thyroid Nodules: A Systematic Review and Meta-Analysis

**PubMed 1558 AND Web of Science 800**

("Artificial Intelligence"[MeSH Terms] OR ("Artificial Intelligence"[MeSH Terms] OR ("artificial"[All Fields] AND "intelligence"[All Fields]) OR "Artificial Intelligence"[All Fields] OR ("intelligence"[All Fields] AND "artificial"[All Fields]) OR "intelligence artificial"[All Fields] OR ("Artificial Intelligence"[MeSH Terms] OR ("artificial"[All Fields] AND "intelligence"[All Fields]) OR "Artificial Intelligence"[All Fields] OR ("computer"[All Fields] AND "reasoning"[All Fields]) OR "computer reasoning"[All Fields]) OR ("Artificial Intelligence"[MeSH Terms] OR ("artificial"[All Fields] AND "intelligence"[All Fields]) OR "Artificial Intelligence"[All Fields] OR ("reasoning"[All Fields] AND "computer"[All Fields]) OR "reasoning computer"[All Fields]) OR (("antagonists and inhibitors"[MeSH Subheading] OR ("antagonists"[All Fields] AND "inhibitors"[All Fields]) OR "antagonists and inhibitors"[All Fields] OR "ai"[All Fields]) AND ("Artificial Intelligence"[MeSH Terms] OR ("artificial"[All Fields] AND "intelligence"[All Fields]) OR "Artificial Intelligence"[All Fields])) OR ("Artificial Intelligence"[MeSH Terms] OR ("artificial"[All Fields] AND "intelligence"[All Fields]) OR "Artificial Intelligence"[All Fields] OR ("machine"[All Fields] AND "intelligence"[All Fields]) OR "machine intelligence"[All Fields]) OR ("Artificial Intelligence"[MeSH Terms] OR ("artificial"[All Fields] AND "intelligence"[All Fields]) OR "Artificial Intelligence"[All Fields] OR ("intelligence"[All Fields] AND "machine"[All Fields]) OR "intelligence machine"[All Fields]) OR ("Artificial Intelligence"[MeSH Terms] OR ("artificial"[All Fields] AND "intelligence"[All Fields]) OR "Artificial Intelligence"[All Fields] OR ("computational"[All Fields] AND "intelligence"[All Fields]) OR "computational intelligence"[All Fields]) OR ("Artificial Intelligence"[MeSH Terms] OR ("artificial"[All Fields] AND "intelligence"[All Fields]) OR "Artificial Intelligence"[All Fields] OR ("intelligence"[All Fields] AND "computational"[All Fields]) OR "intelligence computational"[All Fields]) OR ("Artificial Intelligence"[MeSH Terms] OR ("artificial"[All Fields] AND "intelligence"[All Fields]) OR "Artificial Intelligence"[All Fields] OR ("computer"[All Fields] AND "vision"[All Fields] AND "systems"[All Fields]) OR "computer vision systems"[All Fields]) OR ("Artificial Intelligence"[MeSH Terms] OR ("artificial"[All Fields] AND "intelligence"[All Fields]) OR "Artificial Intelligence"[All Fields] OR ("computer"[All Fields] AND "vision"[All Fields] AND "system"[All Fields]) OR "computer vision system"[All Fields]) OR ("Artificial Intelligence"[MeSH Terms] OR ("artificial"[All Fields] AND "intelligence"[All Fields]) OR "Artificial Intelligence"[All Fields] OR ("system"[All Fields] AND "computer"[All Fields] AND "vision"[All Fields]) OR "system computer vision"[All Fields]) OR ("Artificial Intelligence"[MeSH Terms] OR ("artificial"[All Fields] AND "intelligence"[All Fields]) OR "Artificial Intelligence"[All Fields] OR ("systems"[All Fields] AND "computer"[All Fields] AND "vision"[All Fields]) OR "systems computer vision"[All Fields]) OR ("Artificial Intelligence"[MeSH Terms] OR ("artificial"[All Fields] AND "intelligence"[All Fields]) OR "Artificial Intelligence"[All Fields] OR ("vision"[All Fields] AND "system"[All Fields] AND "computer"[All Fields])) OR ("Artificial Intelligence"[MeSH Terms] OR ("artificial"[All Fields] AND "intelligence"[All Fields]) OR "Artificial Intelligence"[All Fields] OR ("vision"[All Fields] AND "systems"[All Fields] AND "computer"[All Fields])) OR (("education"[MeSH Terms] OR "education"[All Fields] OR ("knowledge"[All Fields] AND "acquisition"[All Fields]) OR "knowledge acquisition"[All Fields]) AND ("computability"[All Fields] OR "computable"[All Fields] OR "computating"[All Fields] OR "computation"[All Fields] OR "computational"[All Fields] OR "computations"[All Fields] OR "compute"[All Fields] OR "computed"[All Fields] OR "computer s"[All Fields] OR "computers"[MeSH Terms] OR "computers"[All Fields] OR "computer"[All Fields] OR "computes"[All Fields] OR "computing"[All Fields] OR "computional"[All Fields])) OR ((("acquisition"[All Fields] OR "acquisitions"[All Fields]) AND ("knowledge"[MeSH Terms] OR "knowledge"[All Fields] OR "knowledge s"[All Fields] OR "knowledgeability"[All Fields] OR "knowledgeable"[All Fields] OR "knowledgeably"[All Fields] OR "knowledges"[All Fields])) AND ("computability"[All Fields] OR "computable"[All Fields] OR "computating"[All Fields] OR "computation"[All Fields] OR "computational"[All Fields] OR "computations"[All Fields] OR "compute"[All Fields] OR "computed"[All Fields] OR "computer s"[All Fields] OR "computers"[MeSH Terms] OR "computers"[All Fields] OR "computer"[All Fields] OR "computes"[All Fields] OR "computing"[All Fields] OR "computional"[All Fields])) OR ((("knowledge"[MeSH Terms] OR "knowledge"[All Fields] OR "knowledge s"[All Fields] OR "knowledgeability"[All Fields] OR "knowledgeable"[All Fields] OR "knowledgeably"[All Fields] OR "knowledges"[All Fields]) AND ("representability"[All Fields] OR "representable"[All Fields] OR "representation"[All Fields] OR "representation s"[All Fields] OR "representational"[All Fields] OR "representations"[All Fields])) AND ("computability"[All Fields] OR "computable"[All Fields] OR "computating"[All Fields] OR "computation"[All Fields] OR "computational"[All Fields] OR "computations"[All Fields] OR "compute"[All Fields] OR "computed"[All Fields] OR "computer s"[All Fields] OR "computers"[MeSH Terms] OR "computers"[All Fields] OR "computer"[All Fields] OR "computes"[All Fields] OR "computing"[All Fields] OR "computional"[All Fields])) OR ((("knowledge"[MeSH Terms] OR "knowledge"[All Fields] OR "knowledge s"[All Fields] OR "knowledgeability"[All Fields] OR "knowledgeable"[All Fields] OR "knowledgeably"[All Fields] OR "knowledges"[All Fields]) AND ("representability"[All Fields] OR "representable"[All Fields] OR "representation"[All Fields] OR "representation s"[All Fields] OR "representational"[All Fields] OR "representations"[All Fields])) AND ("computability"[All Fields] OR "computable"[All Fields] OR "computating"[All Fields] OR "computation"[All Fields] OR "computational"[All Fields] OR "computations"[All Fields] OR "compute"[All Fields] OR "computed"[All Fields] OR "computer s"[All Fields] OR "computers"[MeSH Terms] OR "computers"[All Fields] OR "computer"[All Fields] OR "computes"[All Fields] OR "computing"[All Fields] OR "computional"[All Fields])) OR ((("representability"[All Fields] OR "representable"[All Fields] OR "representation"[All Fields] OR "representation s"[All Fields] OR "representational"[All Fields] OR "representations"[All Fields]) AND ("knowledge"[MeSH Terms] OR "knowledge"[All Fields] OR "knowledge s"[All Fields] OR "knowledgeability"[All Fields] OR "knowledgeable"[All Fields] OR "knowledgeably"[All Fields] OR "knowledges"[All Fields])) AND ("computability"[All Fields] OR "computable"[All Fields] OR "computating"[All Fields] OR "computation"[All Fields] OR "computational"[All Fields] OR "computations"[All Fields] OR "compute"[All Fields] OR "computed"[All Fields] OR "computer s"[All Fields] OR "computers"[MeSH Terms] OR "computers"[All Fields] OR "computer"[All Fields] OR "computes"[All Fields] OR "computing"[All Fields] OR "computional"[All Fields])) OR (("intelligence"[MeSH Terms] OR "intelligence"[All Fields] OR "intelligences"[All Fields] OR "intelligent"[All Fields] OR "intelligently"[All Fields] OR "intelligibilities"[All Fields] OR "intelligibility"[All Fields] OR "intelligible"[All Fields]) AND ("platform"[All Fields] OR "platform s"[All Fields] OR "platforms"[All Fields])) OR ("machine learning"[MeSH Terms] OR ("machine"[All Fields] AND "learning"[All Fields]) OR "machine learning"[All Fields]) OR ("machine learning"[MeSH Terms] OR ("machine"[All Fields] AND "learning"[All Fields]) OR "machine learning"[All Fields] OR ("learning"[All Fields] AND "machine"[All Fields]) OR "learning machine"[All Fields]) OR ("machine learning"[MeSH Terms] OR ("machine"[All Fields] AND "learning"[All Fields]) OR "machine learning"[All Fields] OR ("transfer"[All Fields] AND "learning"[All Fields]) OR "transfer learning"[All Fields]) OR ("machine learning"[MeSH Terms] OR ("machine"[All Fields] AND "learning"[All Fields]) OR "machine learning"[All Fields] OR ("learning"[All Fields] AND "transfer"[All Fields]) OR "learning transfer"[All Fields]) OR (("convolute"[All Fields] OR "convoluted"[All Fields] OR "convolutes"[All Fields] OR "convoluting"[All Fields] OR "convolution"[All Fields] OR "convolutional"[All Fields] OR "convolutions"[All Fields] OR "convolutive"[All Fields]) AND ("neural networks, computer"[MeSH Terms] OR ("neural"[All Fields] AND "networks"[All Fields] AND "computer"[All Fields]) OR "computer neural networks"[All Fields] OR ("neural"[All Fields] AND "network"[All Fields]) OR "neural network"[All Fields])) OR "CNN"[All Fields])) AND ("Thyroid Nodule"[MeSH Terms] OR ("Thyroid Nodule"[MeSH Terms] OR ("thyroid"[All Fields] AND "nodule"[All Fields]) OR "Thyroid Nodule"[All Fields] OR ("nodules"[All Fields] AND "thyroid"[All Fields]) OR "nodules thyroid"[All Fields] OR ("Thyroid Nodule"[MeSH Terms] OR ("thyroid"[All Fields] AND "nodule"[All Fields]) OR "Thyroid Nodule"[All Fields] OR ("nodule"[All Fields] AND "thyroid"[All Fields]) OR "nodule thyroid"[All Fields]) OR ("Thyroid Nodule"[MeSH Terms] OR ("thyroid"[All Fields] AND "nodule"[All Fields]) OR "Thyroid Nodule"[All Fields] OR ("thyroid"[All Fields] AND "nodules"[All Fields]) OR "thyroid nodules"[All Fields])) OR ("Thyroid Neoplasms"[MeSH Terms] OR ("Thyroid Neoplasms"[MeSH Terms] OR ("thyroid"[All Fields] AND "neoplasms"[All Fields]) OR "Thyroid Neoplasms"[All Fields] OR ("neoplasm"[All Fields] AND "thyroid"[All Fields]) OR "neoplasm thyroid"[All Fields] OR ("Thyroid Neoplasms"[MeSH Terms] OR ("thyroid"[All Fields] AND "neoplasms"[All Fields]) OR "Thyroid Neoplasms"[All Fields] OR ("thyroid"[All Fields] AND "neoplasm"[All Fields]) OR "thyroid neoplasm"[All Fields]) OR ("Thyroid Neoplasms"[MeSH Terms] OR ("thyroid"[All Fields] AND "neoplasms"[All Fields]) OR "Thyroid Neoplasms"[All Fields] OR ("neoplasms"[All Fields] AND "thyroid"[All Fields]) OR "neoplasms thyroid"[All Fields]) OR ("Thyroid Neoplasms"[MeSH Terms] OR ("thyroid"[All Fields] AND "neoplasms"[All Fields]) OR "Thyroid Neoplasms"[All Fields] OR ("thyroid"[All Fields] AND "carcinoma"[All Fields]) OR "thyroid carcinoma"[All Fields]) OR ("Thyroid Neoplasms"[MeSH Terms] OR ("thyroid"[All Fields] AND "neoplasms"[All Fields]) OR "Thyroid Neoplasms"[All Fields] OR ("carcinomas"[All Fields] AND "thyroid"[All Fields]) OR "carcinomas thyroid"[All Fields]) OR ("Thyroid Neoplasms"[MeSH Terms] OR ("thyroid"[All Fields] AND "neoplasms"[All Fields]) OR "Thyroid Neoplasms"[All Fields] OR ("carcinoma"[All Fields] AND "thyroid"[All Fields]) OR "carcinoma thyroid"[All Fields]) OR ("Thyroid Neoplasms"[MeSH Terms] OR ("thyroid"[All Fields] AND "neoplasms"[All Fields]) OR "Thyroid Neoplasms"[All Fields] OR ("thyroid"[All Fields] AND "carcinomas"[All Fields]) OR "thyroid carcinomas"[All Fields]) OR ("Thyroid Neoplasms"[MeSH Terms] OR ("thyroid"[All Fields] AND "neoplasms"[All Fields]) OR "Thyroid Neoplasms"[All Fields] OR ("cancer"[All Fields] AND "thyroid"[All Fields]) OR "cancer of the thyroid"[All Fields]) OR ("Thyroid Neoplasms"[MeSH Terms] OR ("thyroid"[All Fields] AND "neoplasms"[All Fields]) OR "Thyroid Neoplasms"[All Fields] OR ("cancer"[All Fields] AND "thyroid"[All Fields]) OR "cancer of thyroid"[All Fields]) OR ("Thyroid Neoplasms"[MeSH Terms] OR ("thyroid"[All Fields] AND "neoplasms"[All Fields]) OR "Thyroid Neoplasms"[All Fields] OR ("thyroid"[All Fields] AND "cancers"[All Fields]) OR "thyroid cancers"[All Fields]) OR ("Thyroid Neoplasms"[MeSH Terms] OR ("thyroid"[All Fields] AND "neoplasms"[All Fields]) OR "Thyroid Neoplasms"[All Fields] OR ("thyroid"[All Fields] AND "cancer"[All Fields]) OR "thyroid cancer"[All Fields]) OR ("Thyroid Neoplasms"[MeSH Terms] OR ("thyroid"[All Fields] AND "neoplasms"[All Fields]) OR "Thyroid Neoplasms"[All Fields] OR ("cancers"[All Fields] AND "thyroid"[All Fields]) OR "cancers thyroid"[All Fields]) OR ("Thyroid Neoplasms"[MeSH Terms] OR ("thyroid"[All Fields] AND "neoplasms"[All Fields]) OR "Thyroid Neoplasms"[All Fields] OR ("cancer"[All Fields] AND "thyroid"[All Fields]) OR "cancer thyroid"[All Fields]) OR ("Thyroid Neoplasms"[MeSH Terms] OR ("thyroid"[All Fields] AND "neoplasms"[All Fields]) OR "Thyroid Neoplasms"[All Fields] OR ("thyroid"[All Fields] AND "adenoma"[All Fields]) OR "thyroid adenoma"[All Fields]) OR ("Thyroid Neoplasms"[MeSH Terms] OR ("thyroid"[All Fields] AND "neoplasms"[All Fields]) OR "Thyroid Neoplasms"[All Fields] OR ("adenomas"[All Fields] AND "thyroid"[All Fields]) OR "adenomas thyroid"[All Fields]) OR ("Thyroid Neoplasms"[MeSH Terms] OR ("thyroid"[All Fields] AND "neoplasms"[All Fields]) OR "Thyroid Neoplasms"[All Fields] OR ("adenoma"[All Fields] AND "thyroid"[All Fields]) OR "adenoma thyroid"[All Fields]) OR ("Thyroid Neoplasms"[MeSH Terms] OR ("thyroid"[All Fields] AND "neoplasms"[All Fields]) OR "Thyroid Neoplasms"[All Fields] OR ("thyroid"[All Fields] AND "adenomas"[All Fields]) OR "thyroid adenomas"[All Fields]))))

**Cochrane Library 34**

#1 (Artificial Intelligence or Intelligence, Artificial or Computer Reasoning or Reasoning, Computer or AI (Artificial Intelligence) or Machine Intelligence or Intelligence, Machine or Computational Intelligence or Intelligence, Computational or Computer Vision Systems or Computer Vision System or System, Computer Vision or Systems, Computer Vision or Vision System, Computer or Vision Systems, Computer or Knowledge Acquisition (Computer) or Acquisition, Knowledge (Computer) or Knowledge Representation (Computer) or Knowledge Representations (Computer) or Representation, Knowledge (Computer) or intelligent platform or Machine Learning or Learning, Machine or Transfer Learning or Learning, Transfer or Convolution Neural Network or CNN):ti,ab,kw (Word variations have been searched) 12231

#2 Thyroid Nodule or Nodules, Thyroid or Nodule, Thyroid or Thyroid Nodules or Thyroid Neoplasms or Neoplasm, Thyroid or Thyroid Neoplasm or Neoplasms, Thyroid or Thyroid Carcinoma or Carcinomas, Thyroid or Carcinoma, Thyroid or Thyroid Carcinomas or Cancer of the Thyroid or Cancer of Thyroid or Thyroid Cancers or Thyroid Cancer or Cancers, Thyroid or Cancer, Thyroid or Thyroid Adenoma or Adenomas, Thyroid or Adenoma, Thyroid or Thyroid Adenomas 3466

#3 #1 and #2 35

Figure S1: Sensitivity and specificity forest plots after simultaneously removing four studies with high heterogeneity
