# Supplementary material for: Diagnostic performance of ultrasound characteristics-based artificial intelligence models for thyroid nodules: a systematic review and meta-analysis
Source: Front Oncol. 2025 Sep 3;15:1614603. doi: 10.3389/fonc.2025.1614603 (PMC12440764; doi:10.3389/fonc.2025.1614603)
Supplement: Supplementary file 2 [file DataSheet2.doc]

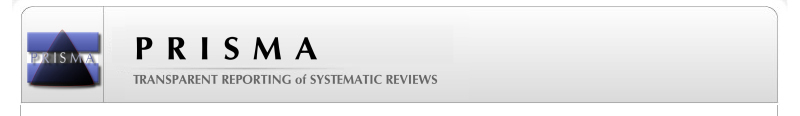
**PRISMA 2009 Flow Diagram**

**Screening**

**Included**

**Eligibility**

**Identification**

Records identified through database searching
(n = 2392 )

Additional records identified through other sources
(n = 0)

Records after duplicates removed
(n = 1772)

Records screened
(n = 239)

Full-text articles assessed for eligibility
(n = 192)

Not matched inclusion criteria (n=66)

Inadequate information
(n =98)

Studies included in qualitative synthesis
(n = 28)

Studies included in quantitative synthesis (meta-analysis)
(n = 28)

Records excluded
(n =1533)

Articles excluded
(n =47)
